# Design of Inhibitors of the Intrinsically Disordered Protein NUPR1: Balance between Drug Affinity and Target Function

**DOI:** 10.3390/biom11101453

**Published:** 2021-10-03

**Authors:** Bruno Rizzuti, Wenjun Lan, Patricia Santofimia-Castaño, Zhengwei Zhou, Adrián Velázquez-Campoy, Olga Abián, Ling Peng, José L. Neira, Yi Xia, Juan L. Iovanna

**Affiliations:** 1 CNR-NANOTEC, SS Rende (CS), Department of Physics, University of Calabria, Via P. Bucci, Cubo 31 C, 87036 Rende, Cosenza, Italy; bruno.rizzuti@cnr.it; 2 Instituto de Biocomputación y Física de Sistemas Complejos, Joint Units IQFR-CSIC-BIFI, and GBsC-CSIC-BIFI, Universidad de Zaragoza, 50018 Zaragoza, Spain; adrianvc@unizar.es (A.V.-C.); oabifra@unizar.es (O.A.); jlneira@umh.es (J.L.N.); 3 Centre de Recherche en Cancérologie de Marseille (CRCM), INSERM U1068, CNRS UMR 7258, Institut Paoli-Calmettes, Aix-Marseille Université, 13288 Marseille, France; wenjun.lan@etu.univ-amu.fr (W.L.); patricia.santofimia@inserm.fr (P.S.-C.); 4 Aix-Marseille Université, CNRS, Centre Interdisciplinaire de Nanoscience de Marseille, UMR 7325, «Equipe Labellisée Ligue Contre le Cancer», 13288 Marseille, France; ling.peng@univ-amu.fr; 5 Chongqing Key Laboratory of Natural Product Synthesis and Drug Research, School of Pharmaceutical Sciences, Chongqing University, Chongqing 401331, China; zhouzhengwei610@126.com; 6 Aragon Institute for Health Research (IIS Aragon), 50009 Zaragoza, Spain; 7 Centro de Investigación Biomédica en Red en el Área Temática de Enfermedades Hepáticas y Digestivas (CIBERehd), 28029 Barcelona, Spain; 8 Departamento de Bioquímica y Biología Molecular y Celular, Universidad de Zaragoza, 50009 Zaragoza, Spain; 9 Fundacion ARAID, Government of Aragon, 50018 Zaragoza, Spain; 10 Instituto Aragonés de Ciencias de la Salud (IACS), 50009 Zaragoza, Spain; 11 IDIBE, Universidad Miguel Hernández, 03202 Elche, Alicante, Spain

**Keywords:** intrinsically disordered proteins, nuclear protein 1, drug discovery, ligand-based design, isothermal titration calorimetry, biological assays

## Abstract

Intrinsically disordered proteins (IDPs) are emerging as attractive drug targets by virtue of their physiological ubiquity and their prevalence in various diseases, including cancer. NUPR1 is an IDP that localizes throughout the whole cell, and is involved in the development and progression of several tumors. We have previously repurposed trifluoperazine (TFP) as a drug targeting NUPR1 and, by using a ligand-based approach, designed the drug ZZW-115 starting from the TFP scaffold. Such derivative compound hinders the development of pancreatic ductal adenocarcinoma (PDAC) in mice, by hampering nuclear translocation of NUPR1. Aiming to further improve the activity of ZZW-115, here we have used an indirect drug design approach to modify its chemical features, by changing the substituent attached to the piperazine ring. As a result, we have synthesized a series of compounds based on the same chemical scaffold. Isothermal titration calorimetry (ITC) showed that, with the exception of the compound preserving the same chemical moiety at the end of the alkyl chain as ZZW-115, an increase of the length by a single methylene group (i.e., ethyl to propyl) significantly decreased the affinity towards NUPR1 measured in vitro, whereas maintaining the same length of the alkyl chain and adding heterocycles favored the binding affinity. However, small improvements of the compound affinity towards NUPR1, as measured by ITC, did not result in a corresponding improvement in their inhibitory properties and in cellulo functions, as proved by measuring three different biological effects: hindrance of the nuclear translocation of the protein, sensitization of cells against DNA damage mediated by NUPR1, and prevention of cancer cell growth. Our findings suggest that a delicate compromise between favoring ligand affinity and controlling protein function may be required to successfully design drugs against NUPR1, and likely other IDPs.

## 1. Introduction

Intrinsically disordered proteins (IDPs) have very high flexibility, hence do not maintain a stable conformation. IDPs are frequently involved in regulatory and signaling processes and their functions include cell cycle control, transcriptional regulation, replication, differentiation, and RNA processing, which are mediated by protein-protein and protein-nucleic acid interactions [1,2,3,4], as well as the formation of membraneless organelles via liquid-liquid phase separation [5]. Furthermore, IDPs are engaged in multivalent and/or promiscuous interactions, and, in fact, proteins involved in several biological pathways have a large proportion of disorder in their sequences [6].

NUPR1 (UniProtKB O60356), or nuclear protein 1, is an 82-residue-long (8 kDa), highly basic IDP, whose proper function is unknown, although it was first described as being activated in the exocrine pancreas in response to the cellular injury induced by pancreatitis [7]. NUPR1 is involved in cell-cycle regulation; in fact, the inducible expression of the *Nupr1* gene has been observed under several stress conditions [8,9]. NUPR1 is over-expressed in almost any, if not all, cancer tissues [8,10,11]. Moreover, it is involved in several protein cascades, such as in the processes regulating apoptosis through interaction with the oncoprotein ProTα [12], DNA repair in combination with the male specific lethal protein 1 [13,14], and cell-differentiation processes together with Polycomb proteins [15]. We have previously shown that genetic inactivation of *Nupr1* antagonizes the growth of pancreatic cancer [16,17]; furthermore, its inactivation stops the growth of hepatocarcinoma [18], cholangiocarcinoma [19], non-small cell lung cancer [20], glioblastoma [21], osteosarcoma [22] and multiple myeloma [23]. Collectively, these results indicate that NUPR1 could be exploited as a target to develop new therapies against cancer, by hindering its protein-protein interactions (PPIs) with other molecular partners. However, NUPR1 as a target for drug discovery is challenging due to its unfolded and dynamic nature.

**Figure 1:**
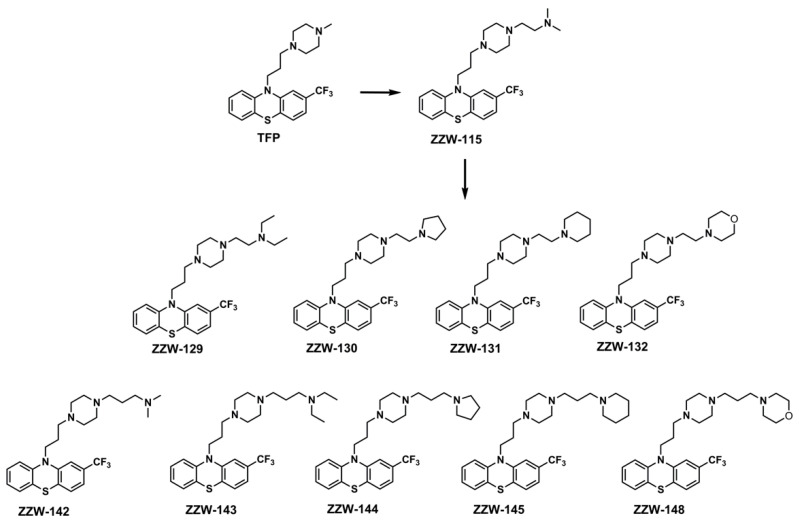
Structures of the ZZW-115-derived compounds used in this work. The structures of TFP and ZZW-115 are also shown for comparison.

We have recently developed a combination of biochemical, biophysical, bioinformatics, and biological approaches for an in vitro, in vivo, in silico, and in cellulo molecular screening, to select potential drug candidates against NUPR1, following a bottom-up approach and a lead-based strategy [24]. We have first repurposed the antipsychotic agent trifluoperazine (TFP) as an anticancer drug candidate, because it completely stops the growth of tumor in human pancreatic cancer xenograft mice [25]. However, TFP also causes neurological effects on treated mice at the doses necessary to obtain its antitumor action, precluding its use to treat cancer in clinics. To overcome these issues and increase TFP anticancer activity, we have explored ligand-based drug design (LBDD) with a rational in silico approach, modifying TFP by adding different functional groups [26]. As a result, we have identified a lead compound, ZZW-115, which bears the substitution of the methyl group in one of the nitrogen atoms of the piperazine group of TFP by an alkylated amine group (Figure 1). ZZW-115 has a far better antitumor activity than TFP and no appreciable side effects in mice. It induces cell death by both necroptotic and apoptotic mechanisms, with a mitochondrial metabolism failure that triggers lower production of ATP and overproduction of reactive oxygen species [26]. We have also demonstrated that ZZW-115 hampers NUPR1 nuclear translocation [27].

The success of identifying the lead compound ZZW-115 against NUPR1 has stimulated us to propose further improvements in the anticancer activity of ZZW-115 by using a similar combined approach as previously pursued. In particular, our molecular optimization strategy falls within the realm of LBDD, also known as indirect drug design [28,29], which is based on the evolution of the molecular structure of compounds with known binding properties against the protein target, despite the absence of the knowledge of a binding site. In principle, even an indirect drug design should completely rely on a rational approach, possibly guided by the identification of a pharmacophore model that summarizes the key intermolecular interactions driving the binding to the specific biological target [30]. However, this is particularly challenging in the case of IDPs, due to subtle dynamic effects that govern the binding of ligands to disordered regions. For this reason, LBDD can be supplemented with a more empirical approach which consists in adding functional groups using synthetic chemical methodologies. Thus, we have used a combination of the two approaches to optimize ZZW-115 with the aim of improving its affinity towards NUPR1 and, then, its anti-cancer activity.

As a result of our strategy, in this work, we have designed and synthesized nine derivatives that maintain the basic scaffold of ZZW-115, but contain larger aromatic or alkylated groups at the distal nitrogen of the piperazine ring (Figure 1). Biophysical experiments in vitro on NUPR1, by using isothermal titration calorimetry (ITC), showed that the affinity of these derived compounds was substituent-dependent and, as a general trend, their binding energy towards NUPR1 varied with the size of the introduced chemical group. However, results obtained from cell experiments—namely, inhibition of nuclear translocation of NUPR1, sensitization of cells against DNA-damage mediated by it, and hindrance of cancer cell growth—indicate that a more favorable affinity in the binding does not necessarily correlate with the biological effects. At the end, the biological effect of a given compound depends on the binding affinity to the primary target, the effective compound concentration at the appropriate location, and the binding affinity to any alternative competing targets present in the complex cell environment. Thus, in designing compounds targeting this IDP, there must be a subtle compromise between increasing drug affinity and altering the protein function, with other properties, such as solubility, crowding, membrane permeation, cellular efflux and cellular metabolism, possibly playing an additional relevant role.

## 2. Materials and Methods

### 2.1. Materials

Ampicillin and isopropyl-β-D-1-tiogalactopyranoside were obtained from Apollo Scientific (Stockport, UK). Imidazole, Trizma base and His-Select HF nickel resin were from Sigma-Aldrich (Madrid, Spain). Triton X-100, and protein marker (PAGEmark Tricolor) were from VWR (Barcelona, Spain). The compound 5-fluorouracile (5-FU) was offered by Institute Paoli-Calmettes. Amicon centrifugal devices with a cut-off molecular weight of 3 kDa were from Millipore (Barcelona, Spain). The rest of the materials were of analytical grade. Water was deionized and purified on a Millipore system. Piperazine analogs, 2-(trifluoromethyl)phenothiazine and other reagents were purchased from Adamas-beta (Shanghai, China) or Energy Chemical (Shanghai, China).

### 2.2. Chemistry: General Methods

All compounds were purified by performing flash chromatography on silica gel (200–300 mesh) or preparative thin-layer chromatography. ^1^H-NMR and ^13^C-NMR spectra were recorded on Agilent DD2 400-MR. The chemical shifts were recorded in parts per million (ppm) with tetramethylsilane as the internal reference. The electrospray ionization mass spectroscopy (ESI-MS) data were acquired on a Waters Acquity SQ Detector mass spectrometer or Finigan LCQ mass spectrometer. The high-resolution spectra of ESI-MS were recorded on Bruker SolariX 7.0 T mass spectrometer or IonSpec 4.7 Tesla Fourier Transform mass spectrometer. All MS analysis samples were prepared as solutions in methanol. Analytical HPLC runs were performed on Waters 1525 using columns packed with Inertsil^®^ ODS-3 5mm 4.6 × 250 mm (method 1) and Inertsil^®^ hypersil C8 4.6×250 mm and (method 2) produced by GL sciences Inc, with 2489 UV/Visible detector, wavelength 254 nm. All the samples were dissolved in methanol (MeOH). The mobile phase consisted of an isocratic elution of CH_3_CN/CH_3_OH (30/70) with 0.1% TFA (trifluoracetic acid). The flow rate was 1.0 mL/min. Temperature was 25 °C.

### 2.3. Protein Expression and Purification

Expression and purification of NUPR1 was carried out as described earlier by using Ni-affinity and gel filtration chromatography [12,13,14,15,27]. The purity of NUPR1 was in all cases larger than 95%, as judged by visual inspection in SDS-PAGE. Protein concentration was determined from the absorbance of the two tyrosines in the sequence of NUPR1 [31]. We have previously shown [25,26] that aggregation of the protein does not occur in the presence of ZZW-115 or its other derivatives; only the binding of NUPR1 to the corresponding compound was observed.

### 2.4. Computational Modelling of ZZW-115-Derived Compounds

The binding of ZZW-115 and derived compounds to NUPR1 was modeled in silico following a procedure previously described [26]. In brief, capped fragments of the protein structure with a length of seven amino acid residues and belonging to the hot-spots of NUPR1 (regions around Ala33 and Thr68 residues) were used as a host for molecular docking of the compounds, performed by using the software AutoDock Vina [32]. The small molecular complexes obtained, mimicking each compound bound to a transient protein binding pocket, were refined in 1 ns molecular dynamics (MD) simulations carried out in the isobaric-isothermal ensemble using the GROMACS simulation package [33]. The force fields used were AMBER ff99SB-ILDN [34] for the protein and GAFF [35] for the compounds, whereas full hydration in explicit solvent was obtained by using the IDP-specific water model TIP4P-D [36]. Other simulation conditions (including modelling of electrostatics and van der Waals interactions, use of thermostat and barostat, and treatment of periodic boundary conditions) were as previously described [37,38]. After equilibration in MD simulation runs, the binding affinity of the compounds was re-evaluated using AutoDock Vina in score-only mode [32].

### 2.5. Organic Synthesis of ZZW-115-Derived Compounds

The ZZW-115-derived compounds were synthesized as described previously [26]. To a solution of compound **a** (50 mg, 0.13 mmol) in dimethyl-formamide (DMF) (3 mL), the corresponding piperazine derivatives (0.26 mmol) were added. The mixture was stirred at room temperature overnight under nitrogen atmosphere in dark. Then, the reaction system was concentrated and the desired product was isolated by column chromatography (CH_2_Cl_2_: MeOH: NH_4_OH = 20:1:0.1). The analytical data of the synthetic ZZW-115 derivatives are presented below:

*ZZW-129*. Colorless wax. HPLC: t_R_ = 5.271 min (Method 1, purity 98.8%), t_R_ = 4.603 min (Method 2, purity > 99%). ^1^H-NMR (400 MHz, CDCl_3_): δ 7.04–7.19 (m, 4H, phenyl-H), 6.97 (s, 1H, phenyl-H), 6.84–6.89 (m, 2H, phenyl-H), 3.88 (t, 2H, *J* = 6.8 Hz, -CH_2_-), 2.36–2.51 (m, 16H, -CH_2_-), 1.83-1.90 (m, 4H, -CH_2_-), 0.96 (t, 6H, *J* = 7.2 Hz, -CH_3_). ^13^C-NMR (100 MHz, CDCl_3_): δ 145.68, 144.28, 129.87, 129.37, 127, 60, 127.51, 127.40, 124.00, 123.41, 118.99, 115.89, 111.89, 56.33, 55.40, 53.65, 53.18, 49.96, 47.32, 45.31, 29.69. MS (ESI, *m*/*z*): 492.26 [M + H]^+^. HRMS: calcd for C_26_H_35_F_3_N_4_S, [M + H]^+^, 493.2535; found, 493.2619.

*ZZW-130*. Colorless wax. HPLC: t_R_ = 5.321 min (Method 1, purity > 99%), t_R_ = 4.637 min (Method 2, purity > 99%). ^1^H-NMR (400 MHz, CDCl_3_): δ 7.10–7.19 (m, 4H, phenyl-H), 7.03 (s, 1H, phenyl-H), 6.90–6.96 (m, 2H, phenyl-H), 3.95 (t, 2H, *J* = 6.8 Hz, -CH_2_-), 2.45–2.62 (m, 18H, -CH_2_-), 1.90–1.97 (m, 2H, -CH_2_-), 1.77 (s, 4H, -CH_2_-). ^13^C-NMR (100 MHz, CDCl_3_): δ 145.64, 144.83, 137.41, 127.48, 127.17, 125.09, 122.06, 120.64, 115.70, 114.61, 114.53, 56.82, 56.62, 53.60, 53.16, 45.93, 45.81, 45.28, 24.33, 16.53, 16.39. MS (ESI, *m*/*z*): 491.53 [M + H]^+^. HRMS: calcd for C_26_H_33_F_3_N_4_S, [M + H]^+^, 491.2378; found, 443.2443.

*ZZW-131*. Colorless wax. HPLC: t_R_ = 5.907 min (Method 1, purity > 99%), t_R_ = 4.444 min (Method 2, purity > 99%). ^1^H-NMR (400 MHz, CDCl_3_): δ 7.09–7.18 (m, 4H, phenyl-H), 7.03 (s, 1H, phenyl-H), 6.90–6.94 (m, 2H, phenyl-H), 3.94 (t, 2H, *J* = 8.4 Hz, -CH_2_-), 2.40–2.51 (m, 18H, -CH_2_-), 1.90–1.95 (m, 2H, -CH_2_-), 1.55–1.59 (m, 4H, -CH_2_-), 1.42 (s, 2H, -CH_2_-). ^13^C-NMR (100 MHz, CDCl_3_): δ 145.65, 144.26, 129.83, 129.62, 129.41, 127.55, 127.45, 127.34, 123.96, 123.22, 122.99, 118.90, 115.86, 111.82, 56.60, 55.96, 55.38, 55.03, 53.62, 53.23, 45.31, 25.92, 24.30, 24.12. MS (ESI, *m*/*z*): 505.54 [M + H]^+^. HRMS: calcd for C_27_H_35_F_3_N_4_S, [M + H]^+^, 505.2535; found, 505.2599.

*ZZW-132*. Colorless wax. HPLC: t_R_ = 4.844 min (Method 1, purity 98.9%), t_R_ = 4.218 min (Method 2, purity > 99%). ^1^H-NMR (400 MHz, CDCl_3_): δ 7.09–7.18 (m, 4H, phenyl-H), 7.03 (s, 1H, phenyl-H), 6.89–6.95 (m, 2H, phenyl-H), 3.94 (t, 2H, *J* = 6.8 Hz, -CH_2_-), 3.69 (t, 4H, *J* = 4.4 Hz, -CH_2_-), 2.45–2.49 (m, 18H, -CH_2_-), 1.89–1.96 (m, 2H, -CH_2_-). ^13^C-NMR (100 MHz, CDCl_3_): δ 145.63, 144.22, 129.82, 129.64, 129.32, 127.53, 127.44, 127.33, 125.46, 123.94, 122.98, 118.91, 118.87, 115.83, 111.83, 111.79, 66.87, 56.28, 55.53, 55.30, 54.07, 53.57, 53.15, 45.25, 24.07. MS (ESI, *m*/*z*): 507.51 [M + H]^+^. HRMS: calcd for C_26_H_33_F_3_N_4_S, [M + H]^+^, 507.2327; found, 507.2399.

*ZZW-142*. Colorless wax. HPLC: t_R_ = 5.667 min (Method 1, purity > 99%), t_R_ = 4.936 min (Method 2, purity > 99%). ^1^H-NMR (400 MHz, CDCl_3_): δ 7.20–7.10 (m, 4H, phenyl-H), 7.03 (s, 1H, phenyl-H), 6.96–6.91 (m, 2H, phenyl-H), 3.95 (t, 2H, *J* = 7.0 Hz, -CH_2_-), 2.49–2.25 (m, 14H, -CH_2_-), 2.21 (s, 6H, -CH_3_), 1.97–1.90 (m, 2H, -CH_2_-), 1.69–1.61 (m, 2H, -CH_2_-). ^13^C-NMR (100 MHz, CDCl_3_): δ 145.64, 144.21, 129.86, 129.64, 127.58, 127.48, 127.38, 123.97, 123.03, 118.96. 118.92, 115.87, 111.85, 111.82, 57.37, 56.06, 55.28, 53.04, 53.01, 45.23, 44.78, 29.66, 29.28, 24.07, 24.03. MS (ESI, *m*/*z*): 478.24 [M + H]^+^. HRMS: calcd for C_25_H_33_F_3_N_4_S, [M + H]^+^, 479.2378; found, 479.2475.

*ZZW-143*. Colorless wax. HPLC: t_R_ = 5.264 min (Method 1, purity > 99%), t_R_ = 4.371 min (Method 2, purity 96.6%). ^1^H-NMR (400 MHz, CDCl_3_): δ 6.92–7.20 (m, 7H, phenyl-H), 3.87 (t, 2H, *J* = 6.8 Hz, -CH_2_-), 2.22–2.50 (m, 18H, -CH_2_-), 1.84–1.89 (m, 2H, -CH_2_-), 1.53–1.61 (m, 2H, -CH_2_-), 0.95 (t, 6H, *J* = 6.8 Hz, -CH_3_). ^13^C-NMR (100 MHz, CDCl_3_): δ 145.69, 144.29, 129.88, 129.39, 127.58, 127.48, 127.37, 124.01, 123.02, 118.96, 115.89, 111.88, 111.84, 56.68, 55.41, 53.27, 53.22, 50.74, 46.80, 45.36, 29.66, 24.16, 11.34. MS (ESI, *m*/*z*): 507.26 [M + H]^+^. HRMS: calcd for C_27_H_37_F_3_N_4_S, [M + H]^+^, 507.2691; found, 507.2766.

*ZZW-144*. Colorless wax. HPLC: t_R_ = 5.314 min (Method 1, purity 98.9%), t_R_ = 4.641 min (Method 2, purity 97.5%). ^1^H-NMR (400 MHz, CDCl_3_): δ 6.90–7.20 (m, 7H, phenyl-H), 3.95 (t, 2H, *J* = 6.8 Hz, -CH_2_-), 2.80–2.96 (m, 4H, -CH_2_-), 2.36–2.49 (m, 8H, -CH_2_-), 1.81–1.96 (m, 18H, -CH_2_-). ^13^C-NMR (100 MHz, CDCl_3_): δ 145.69, 144.30, 129.88, 129.72, 129.40, 127.58, 127.49, 127.37, 124.01, 123.02, 118.96, 118.93, 115.89, 111.86, 111.85, 56.81, 55.42, 54.65, 54.20, 53.28, 53.23, 45.37, 32.63, 29.67, 26.48, 24.17, 23.39. MS (ESI, *m*/*z*): 505.24 [M + H]^+^. HRMS: calcd for C_27_H_35_F_3_N_4_S, [M + H]^+^, 505.2535; found, 505.2598.

*ZZW-145*. Colorless wax. HPLC: t_R_ = 5.088 min (Method 1, purity 97.2%), t_R_ = 4.434 min (Method 2, purity 97.2%). ^1^H-NMR (400 MHz, CDCl_3_): δ 6.90–7.20 (m, 7H, phenyl-H), 3.95 (t, 2H, *J* = 6.8 Hz, -CH_2_-), 2.30–2.49 (m, 14H, -CH_2_-), 1.92–1.97 (m, 6H, -CH_2_-), 1.70–1.74 (m, 2H, -CH_2_-), 1.60–1.65 (m, 4H, -CH_2_-), 1.43–1.47 (m, 2H, -CH_2_-). ^13^C-NMR (100 MHz, CDCl_3_): δ 145.67, 144.27, 129.85, 129.67, 129.37, 127.56, 127.46, 127.34, 125.48, 123.98, 122.99, 122.78, 118.93, 118.89, 115.87, 111.86, 111.82, 57.34, 56.71, 55.37, 54.50, 53.24, 53.17, 45.33, 29.65, 25.78, 24.32, 24.20, 24.14. MS (ESI, *m*/*z*): 519.51 [M + H]^+^. HRMS: calcd for C_28_H_37_F_3_N_4_S, [M + H]^+^, 519.2691; found, 519.2763.

*ZZW-148*. Colorless wax. HPLC: t_R_ = 4.841 min (Method 1, purity 98.6%), t_R_ = 4.897 min (Method 2, purity > 99%). ^1^H-NMR (400 MHz, CDCl_3_): δ 7.01–7.10 (m, 4H, phenyl-H), 6.95 (s, 1H, phenyl-H), 6.82–6.87 (m, 2H, phenyl-H), 3.87 (t, 2H, *J* = 6.8 Hz, -CH_2_-), 3.62 (d, 4H, *J* = 4.8 Hz, -CH_2_-), 2.26-2.41 (m, 18H, -CH_2_-), 1.82–1.88 (m, 2H, -CH_2_-), 1.56–1.63 (m, 2H, -CH_2_-). ^13^C-NMR (100 MHz, CDCl_3_): δ 145.64, 144.21, 129.84, 127.54, 127.44, 127.33, 123.96, 122.99, 118.91, 118.87, 115.84, 111.84, 111.80, 66.88, 56.97, 56.47, 55.27, 53.67, 53.12, 53.08, 45.26, 29.62, 24.07, 23.85. MS (ESI, *m*/*z*): 521.66 [M + H]^+^. HRMS: calcd for C_27_H_35_F_3_N_4_OS, [M + H]^+^, 521.2484; found, 521.2564.

### 2.6. Cell Production and Viability Assays

Primary pancreatic cancer cells PDAC001T, PDAC012T, PDAC021T, PDAC081T, PDAC082T, PDAC087T, PDAC088T, PDAC089T, and PDAC115T were obtained from the PaCaOmics clinical trial registered at www.clinicaltrials.gov (accessed on 2 October 2021) with registration number NCT01692873. We included PDAC samples from both echoendoscopic ultrasound-guided fine-needle (EUS-FNA) biopsies for patients with unresectable tumors and from surgical specimens for patients undergoing surgery. The tumor samples of these patients were used to generate patient-derived xenograft in nude mice as previously reported [39]. Xenografts obtained from mice were split into several small pieces and used for cell culture in a biosafety chamber: after fine mincing, they were treated with collagenase type V (ref C9263; Sigma-Aldrich, St. Louis, MO, USA) and trypsin/EDTA (ref 25200-056; Gibco, Life Technologies, Grand Island, NY, USA) and were suspended in Dulbecco’s modified Eagle’s medium supplemented with 1% *w*/*w* penicillin/streptomycin (Gibco, Life Technologies, Paisley, UK) and 10% fetal bovine serum (Lonza Inc., Walkersville, MD, USA). After centrifugation, cells were resuspended in serum-free ductal media adapted from Schreiber et al. [40] at 37 °C in a 5% CO_2_ incubator. Amplified cells were stored in liquid nitrogen as previously reported [41]. Cells were weaned from antibiotics for ≥48 h before testing.

Cell viability was evaluated in ten pancreatic cancer cell lines, including MiaPaCa-2, and nine primary pancreatic cancer-derived cell lines: PDAC001T, PDAC012T, PDAC021T, PDAC081T, PDAC082T, PDAC087T, PDAC088T, PDAC089T, and PDAC115T. MiaPaCa-2 cells were obtained from ATCC (Manassas, VA, USA) and maintained in DMEM (Invitrogen, Cergy-Pontoise, France), supplemented with 10% phosphate buffer solution (PBS) at 37 °C with 5% CO_2_. The primary pancreatic cancer-derived cells were cultured in serum free ductal media (SFDM) following a procedure adapted from those previously described [40], without antibiotic, and incubated at 37 °C in a 5% CO_2_ incubator. Cells were plated in 96-well plates (5000 cells/well) overnight. Then, the media were supplemented with the compounds to be tested at 0–100 µM concentration, and the samples were incubated for another additional 72 h before performing the measurement. Cell viability was estimated after addition of PrestoBlue™ reagent (Life Technologies, Paris, France) for 3 h according to the PrestoBlue™ cell viability reagent protocol provided by the supplier. Cell viability was normalized when comparing to untreated cell rates. Experiments were performed in triplicate and each set was repeated three times, with similar results.

### 2.7. Isothermal Titration Calorimetry (ITC)

The binding of the ZZW-115-derived compounds to NUPR1 was determined as described [26] by using a high sensitivity isothermal titration calorimeter Auto-iTC200 (MicroCal, Malvern-Panalytical, Malvern, UK). Protein samples and solutions were properly degassed. Experiments were performed with freshly prepared protein solutions at 25 °C. A solution of NUPR1 (20 μM, in sodium phosphate 20 mM, pH 7.0, 2% DMSO) in the calorimetric cell was titrated with a solution of each compound (200 μM, in sodium phosphate 20 mM pH 7.0, 2% DMSO). The inhibitors were dissolved in the same buffer used for the protein. A standard protocol was employed: 19 titrant injections with 2 μL compound solution were programmed with a time spacing of 150 s, a stirring speed of 750 rpm, and a reference power of 10 μcal/s. The heat evolved after each ligand injection was calculated from the integral of the calorimetric signal. The heat due to the binding reaction was obtained as the difference between the reaction heat and the corresponding heat of injection, the latter estimated as a constant value throughout the experiment, and included as an adjustable parameter in the analysis. Control experiments (with the compounds injected into the buffer) were performed under the same experimental conditions.

The association constant (*K*_a_), the enthalpy change (Δ*H*) and the stoichiometry (n) of the binding reaction were obtained through nonlinear regression analysis of experimental data to a model assuming a single ligand binding site for the protein. Experiments were performed in replicate and data were analyzed using in-house-developed software implemented in Origin 7 (OriginLab).

### 2.8. Immunofluorescence Staining

Cells (100000 cells/well) were seeded in 12-well plates on coverslips overnight and then treated with 3 μM concentration of each compound for 24 h. After fixation with 4% paraformaldehyde (Sigma-Aldrich, St. Quentin, Fallavier, France) for 15 min, cells were washed twice with 1x PBS and incubated 1 h with the following antibodies at 1:200 dilution: rabbit anti-NUPR1 primary antibody (homemade) or γH2AX primary antibody (ab26350, Abcam, Paris, France). After the washing steps, samples were incubated 1 h with secondary antibodies at 1:500 dilution (goat anti-rabbit Alexa Fluor 488, A27034, or goat anti-mouse Alexa Fluor 488, A28175, both from Thermo Fisher Scientific, Franklin, MA, USA). ProLong™ Gold Antifade Mountant with DAPI (P36931, Thermo Fisher Scientific) was used to stain the nucleus and seal the samples. Image acquisition of Alexa Fluor 488-derived fluorescence and DAPI staining was detected with an LSM 880 controlled by Zeiss Zen Black 63x lens. Co-localization analysis and measurements in both channels were performed by using the ImageJ Coloc 2 plugin (version 3.0.5).

### 2.9. Genotoxicity Evaluation for Compounds Combination

The 5-FU-triggered DNA damage enhanced by ZZW-115-derived compounds was evaluated by counting the number of γH2AX foci present in each cell nucleus after immunofluorescence staining. MiaPaCa-2 cells were treated with 5-FU (10 μM), alone or in combination with the corresponding ZZW-115-derived compounds (1.5 μM), and the DNA damages caused were quantified after 12 h incubation. Untreated cells were used as a negative control.

### 2.10. Statistics

Statistical analyses were performed by using the unpaired two-tailed Student t test or one-way ANOVA with Tukey’s *post hoc* test. Values are expressed as mean ± SEM (standard deviation of measurements). Data are representative of at least three independent experiments with triplicates completed. A value of P lower than 0.05 was considered significant.

## 3. Results

### 3.1. Docking of Compounds to a Simulated Binding Pocket of NUPR1

A computational modelling of ZZW-115-derived compounds was carried out by using a ligand-based approach, following the same protocol we have used in our previous endeavor leading to the development of ZZW-115 and other related analogs [26]. Molecular docking can reveal the anchoring of drugs to the binding hot spots of NUPR1 in a blind search on the whole simulated protein structure, as already demonstrated for TFP [25]. However, to reduce the conformational search in the identification of new compounds, it is convenient to consider fragments encompassing seven amino acid residues centered around the key protein residues Ala33 and Thr68, as previously reported [26]. These regions have been described to be at the center of the hot spots of the proteins, on the basis of two main pieces of evidence: mutational studies, and in silico screening of different compounds [15,26]. These residues also belong to the most hydrophobic regions of the protein [25]. This methodology is supported by the observation that short-range interactions, and especially local hydrophobicity, are crucial to model the binding in NUPR1 [25,26,42].

Small modifications in the molecular structure of ZZW-115 were tested by inserting the new compound within the protein fragments by molecular docking, and considering the most favorable binding modes obtained. These small complexes, each mimicking a different conformation of a transient binding pocket, were further equilibrated in MD simulations. At the end, the binding energy of the compound was obtained by evaluating it with the scoring function of AutoDock Vina [32], and averaged on the various conformations. The calculated binding scores were compared with the one obtained for ZZW-115, −7.5 ± 0.3 kcal/mol, which is largely due to the molecular scaffold of TFP (–7.0 kcal/mol) [25].

**Figure 2:**
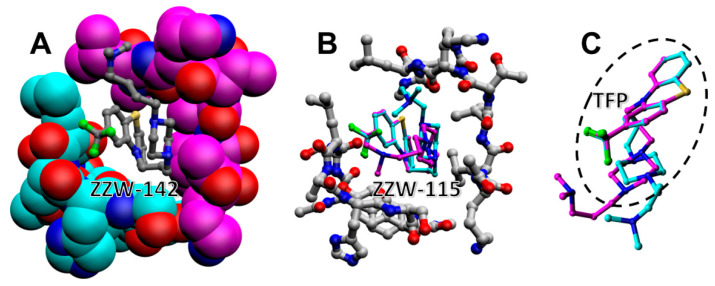
Docking of compounds into the simulated binding pocket of NUPR1: (**A**) Compound ZZW-142 bound into an example of transient pocket formed by the 7-residue regions of NUPR1 centered around Ala33 (cyan) and Thr68 (magenta). (**B**) Cross-docking of compound ZZW-115 into the same binding pocket, showing two different conformations (cyan and magenta). (**C**) Details of the two conformations of ZZW-115 from a different orientation, highlighting the TFP scaffold and the differences in the alkylated tail conformation.

The results showed that variations in the propyl linker between the phenothiazine and piperazine ring did not increase the binding affinity. In contrast, addition of a methylene group in the linker between the piperazine ring and the alkylated amine group (leading to compound ZZW-142; see Figure 1) improved the average binding score by –0.3 kcal/mol with respect to the parent compound ZZW-115. The same variations in the affinity were obtained by cross-docking ZZW-142 into the equilibrated binding pockets containing ZZW-115, or *vice versa*. As shown in Figure 2, differences were due to the arrangement in the conformation of the alkylated chains of the two compounds, while the position of the scaffold remained essentially unchanged. Results were not equally favorable when methylene groups were added in a distal position with respect to the amine group of ZZW-115 (as in compound ZZW-129), because a slightly worse binding energy was obtained (+0.1 kcal/mol).

It is worth to note that the small energetic differences obtained are close to the uncertainty in the evaluation provided by the empiric scoring function, which is on the order of 0.1–0.2 kcal/mol [43]. More importantly, as regards the protein pockets used to model the binding: (i) they have a larger conformational variety (or “fuzziness”, in the terminology commonly used for IDPs) compared to well-defined binding sites, such as those that could be obtained from experimental techniques for well-folded proteins (e.g., X-ray crystallography or NMR) or by homology modelling; and (ii) they cannot be claimed to span an accurate statistical ensemble, whose determination is beyond the current possibilities of both experimental and theoretical methods. These factors restrain the predictive power of the computational techniques and, overall, demonstrate the limitation of LBDD in helping to design IDP inhibitors. In particular, no reliable predictions could be obtained when bulkier chemical moieties were added (as in compounds ZZW-130, ZZW-131 and ZZW-132), because uncertainties in the determination of the binding score were too large (on the order of 1 kcal/mol), and the alkylated tail became too dissimilar to allow a direct comparison with ZZW-115 by using cross-docking.

### 3.2. Synthesis of the ZZW-115-Derived Compounds

We have designed nine 10-(3-(4-ethylpiperazin-1-yl) propyl-10H-phenotiazine derivatives, all modified with different groups on the ethylpiperazine chain. Similar to the synthesis of ZZW-115 [26], the reaction between phenothiazine compound **a** and various piperazine derivatives could produce the ZZW-115-derived compounds in a yield that ranged from 60% to 80% (Scheme S1). The chemical structures and the purities of these compounds were characterized by ^1^H-NMR, ^13^C-NMR, MS and HPLC.

### 3.3. Isothermal Titration Calorimetry in the Presence of ZZW-115-Derived Compounds

**Figure 3:**
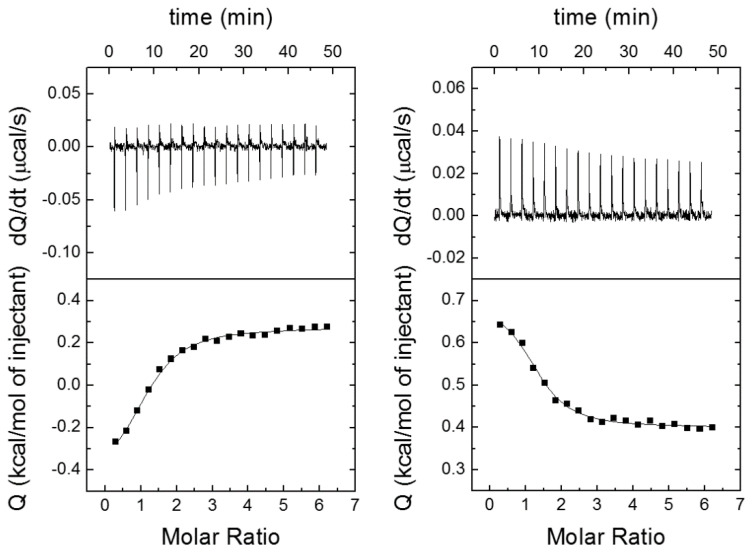
Calorimetric titrations corresponding to the interaction of ZZW-129 (left) and ZZW-142 (right) with NUPR1: Thermograms (thermal power as a function of time) are shown on the top, and binding isotherms (ligand-normalized heat effects as a function of molar ratio) are shown on the bottom. Experiments were performed at 25 °C in sodium phosphate 20 mM, pH 7, 2% DMSO, with 20 μM NUPR1 in the calorimetric cell and 200 μM compound in the titrating syringe, using an Auto-iTC200 instrument (MicroCal-Malvern Panalytical).

The interaction between ZZW-115-Derived compounds and NUPR1 was studied by using ITC, the gold-standard technique in binding-affinity determination, to test our theoretical prediction. The affinity, enthalpy, and stoichiometry of binding were determined at 25 °C. From the thermogram (thermal power as a function of time), the binding isotherm (ligand-normalized heat as a function of the molar ratio) was obtained through integration of the individual heat effect associated with injection of each ligand solution (Figure 3). We used a nonlinear least-squares regression analysis employing a model that considered a single binding site in NUPR1 to estimate binding parameters such as the dissociation constant and the binding enthalpy.

All compounds exhibited dissociation constants in the low micromolar range (Table 1). The interaction is dominated by the entropic contribution, whereas the enthalpic contribution is negligible; all the ΔH values are within a very narrow range of 1 kcal/mol, and with either a negative or positive sign that may depending on small differences upon the mode of interaction. In such a situation, the background heat effect for the injection is large, because even a small difference in DMSO concentration largely modulates this quantity. Nevertheless, this effect does not dominate the thermogram and the interaction parameters could be accurately estimated from the fits of the ITC curves (see again Figure 3). The affinity data do not show in general a clear trend, although it can be noted that the most favorable values have the largest entropic contributions.

Among the compounds tested, ZZW-130 and ZZW-132 were the strongest binders, with a dissociation constant close to 1.7 µM (Figure 3), slightly smaller than that of ZZW-115. These results suggest that the addition of a heterocyclic ring at the terminus of the ethylene moiety hanging from the piperazine ring does not affect the binding affinity of the compounds to NUPR1. That is, the protein is flexible enough to accommodate a bulky ring around its hot spot regions.

In contrast, the affinity constants changed dramatically when the chain hanging from the piperazine ring was enlarged from an ethyl to a propyl group (i.e., a methylene moiety was added) and, in addition, a large ring was added at the terminal end of the new propyl. For instance, ZZW-142 had slightly more favorable affinity for NUPR1 (*K*_d_ = 2.0 µM) than the corresponding parental compound, ZZW-115 (Table 1), suggesting that the addition of a methylene only slightly affected the binding to NUPR1. However, when a heterocyclic ring was incorporated to the terminus of the propyl group, the affinity decreased (Table 1). It is likely that the addition of the propyl group, together with the incorporation of the heterocyclic ring, cannot be accommodated by NUPR1 due to the larger size of the resulting compound. Thus, compounds ZZW-143, ZZW-144, ZZW-145, and ZZW-148 displayed the weakest affinity for NUPR1. The very similar interaction enthalpy observed for most of the compounds suggest that the loss in binding affinity for those containing a longer propyl chain may be related to a conformational entropy loss from the compound stemming from conformationally constraining an additional rotatable bond. This observation is in agreement with the results of our molecular simulations, which showed that the scaffold of TFP essentially dictates the binding affinity of the various ZZW-115-derived compounds, and contributes to explain the difficulties in predicting in silico the fine details of the binding of these ligands, because entropic quantities are notoriously more complex to obtain compared to enthalpic ones by using computational techniques.

### 3.4. Effects of ZZW-115-Derived Compounds on Nuclear Translocation of NUPR1

**Figure 4:**
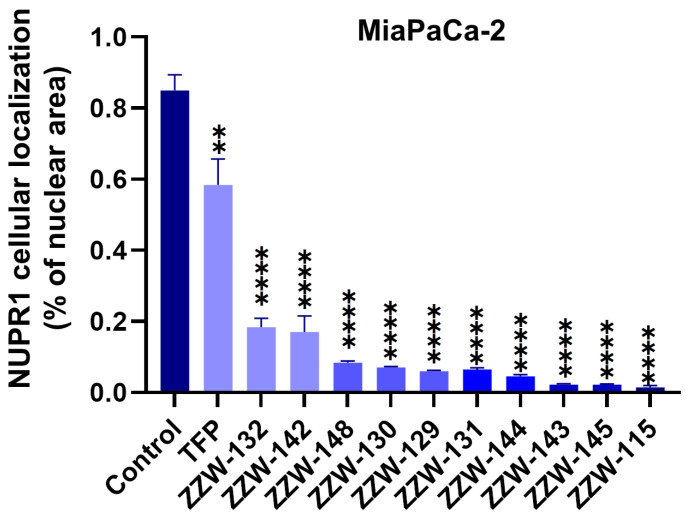
ZZW-115-derived compounds inhibited NUPR1 nuclear translocation. MiaPaCa-2 cells were treated with representative ZZW-115-derived compounds (3 µM) for 6 h. Immunofluorescence with rabbit anti-NUPR1 primary antibody and Alexa 488–labeled goat anti-rabbit secondary antibody were used to reveal the localization of the protein (n = 3). DAPI staining was used to detect cell nuclei, and it was combined with the Alexa 488 fluorescence in the merged panel. The *y*-axis indicates the fraction of area within the nucleus where the presence of NUPR1 was observed. ** *p* < 0.01; **** *p* < 0.0001 (1-way ANOVA, Tukey’s post hoc test).

In our previous work, we have shown that treatment with ZZW-115 inhibited almost completely the translocation of NUPR1 from the cytoplasm to the nucleus by competing with importins in binding their nuclear localization sequence [27]. Here, we observed that all the ZZW-115-derived compounds were capable of hampering NUPR1 nuclear translocation with a higher efficiency than TFP (Figure 4). ZZW-143 and ZZW-145 had an ability to arrest NUPR1 nuclear translocation similar to that of ZZW-115, followed by the efficiency of ZZW-144. This result is somewhat surprising, as compounds ZZW-143 and ZZW-145 had a weaker affinity in vitro for NUPR1 than the rest of the compounds (Table 1). The other ZZW-115-derived compounds had a lower capacity to hamper NUPR1 translocation (Figure 4 and Figure S1). In particular, the worst effect in hampering the nuclear translocation efficiency corresponded to compounds ZZW-132 and ZZW-142.

### 3.5. Treatment with ZZW-115-Derived Compounds Sensitizes Cancer Cells to Genotoxic-induced DNA Damage

**Figure 5:**
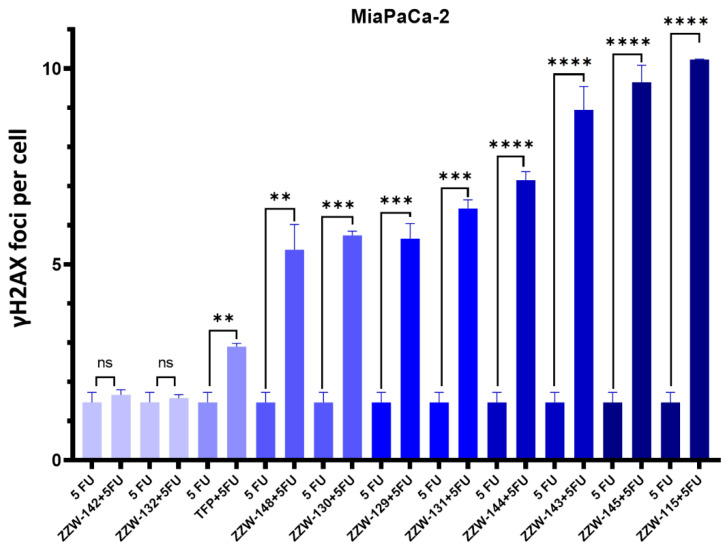
NUPR1 inhibition by ZZW-115-derived compounds potentiated the efficacy of 5-FU in MiaPaCa-2 cells. The efficacy of 5-FU to generate DNA breaks in primary PDAC cells and the boosting effect of ZZW-115 was evaluated by γH2AX immunofluorescence staining. Quantifications of 3 independent experiments were used to evaluate the statistical significance, and they are shown as graphics. ** *p* < 0.01; *** *p*< 0.001; **** *p* < 0.0001 (1-way ANOVA, Tukey’s post hoc test). Data represent mean ± SEM, n = 3.

As NUPR1 is involved in DNA-damage stimulus processes [13,27], we had shown in our previous work that treatment with ZZW-115 sensitizes cancer cells to genotoxic-induced DNA damage [27]. Hence, we hypothesized that ZZW-115-derived compounds could have a similar effect. However, to our surprise, we observed that the ZZW-115-derived compounds showed lower improvement in sensitizing cancer cells to DNA damage induced by 5-FU than the original compound ZZW-115, as indicated by the decrease in the number of foci per cell observed in our experiments (Figure 5 and Figure S2). The sole compound showing a sensitizing effect similar to ZZW-115 was ZZW-145 (which had one of the lowest affinities for NUPR1 in vitro, Table 1). On the other hand, the worst improvement in sensitizing cancer cells to 5-FU occurred for compounds ZZW-132 and ZZW-142 (Figure 5), which showed results similar to the effect caused by the sole action of 5-FU. Therefore, the smallest sensitizing effect was observed for the same two compounds hampering nuclear translocation of NUPR1.

### 3.6. Effects of ZZW-115-Derived Compounds on Pancreatic Cancer Cell Growth

**Figure 6:**
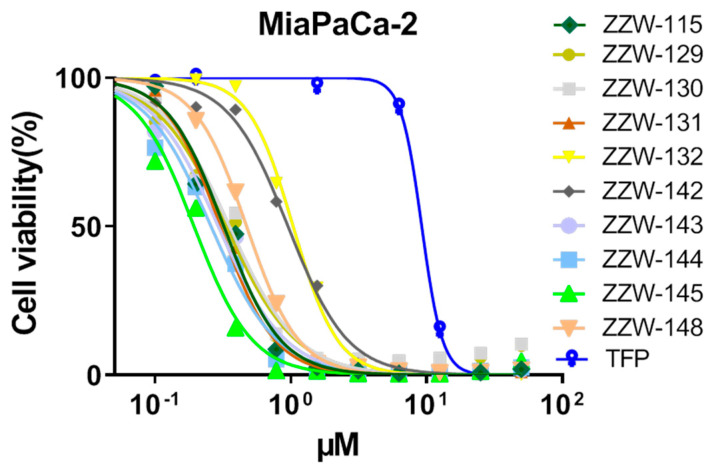
ZZW-115-derived compounds have antitumoral effect: Viability of MiaPaCa-2 cells upon a treatment for 72 h with the ZZW-115-derived compounds.

Treatment of MiaPaCa-2 (a traditional cell line), PDAC001T, PDAC021T, PDAC087T and PDAC115T (basal subtype), PDAC089T (derived from a liver metastasis), and PDAC012T, PDAC081T, PDAC082T, PDAC088T (classical subtype) cells with the ZZW-115-derived compounds showed different effects on the growth of pancreatic cancer cells (Table 2). The results were compared with those previously obtained by TFP and ZZW-115 [26], used as controls. ZZW-115 and most of its derived compounds were found to be 3 to 10 times more efficient in killing cancer cells than the treatment with TFP (and other TFP-derived compounds [26]) in MiaPaCa-2 cells (Figure 6, Table 2). However, most of the compounds showed values of IC_50_ similar to that of ZZW-115 in MiaPaCa-2 cells. Only ZZW-132 and ZZW-142 showed worse activity (having IC_50_ values in the range of 1 µM), although the affinities of both observed in vitro were similar to that of ZZW-115 (Table 1). Taken together, the overall results obtained with MiaPaCa-2 cells demonstrate that the chemical modifications introduced did not seem to increase appreciably the anticancer activity when compared to that of ZZW-115, except for two compounds. More importantly, we did not observe any direct relationship between the affinity measured in vitro by ITC and the anticancer activity of the same compound, neither, in general terms, among the effectivity of the drugs in the different biological activities measured.

For the rest of the assayed cells (Table 2), in broad terms, most of the IC_50_ values were similar to those of ZZW-115, and lower than those of TFP (i.e., they had a better anti-cancer activity). Only ZZW-132 (as it happened with MiaPaca-2 cells) and ZZW-142 were found to show worse IC_50_ values. These results are especially surprising for ZZW-132, as its affinity measured in vitro by ITC was among the most favorable (Table 1).

## 4. Discussion

In this work, we sought to use an indirect design to obtain new compounds targeting the totally unstructured tumorigenic protein NUPR1, building on former successful efforts that had led to the identification of the anti-cancer drug ZZW-115 [24]. Our attempts to further modify this compound were based on previous results obtained starting from the chemical scaffold of TFP, which has the ability to bind NUPR1 [25], and on the chemical features and biological effects of ZZW-115 and other TFP-derived analogs [26]. In our previous studies, we had also used NMR to further characterize the binding of ZZW-115, TFP, and other compounds; however, such technique always identified small variations in the intensity of peaks of residues around the above mentioned hot spots [25,26], which were in complete agreement with the corresponding MD simulations. Such previous accord between MD and NMR studies has prompted us to use only the former technique in the present study on the derivatives of ZZW-115.

We could not develop inhibitors more effective than ZZW-115 due the difficulties in obtaining reliable results by using solely a rational LBDD approach. In fact, the differences obtained by adding in silico small chemical moieties, such as single methylene group in the alkyl chain attached to the piperazine ring of TFP, resulted in very small modifications of the binding affinity, as obtained from the simulation experiments. Moreover, for larger chemical moieties the comparison with ZZW-115 became unclear, and no reliable result could be obtained. Furthermore, at this stage we cannot unambiguously rule out the possibility of binding of any of the compounds to other proteins or other biomolecules. However, studies carried out with previous compounds (TFP, ZZW-115 and its first series of derivatives) on NUPR1-deficient mice did not result in the inhibition of cancer growth [25,26], suggesting that the main target for these drugs is in fact NUPR1.

The modifications introduced to obtain ZZW-115 derivatives were tested by ITC measurements in vitro (Table 1). It was observed that a larger affinity (i.e., smaller dissociation constant) generally corresponded to compounds in which a smaller moiety with an ethylene chain was present in the piperazine ring of the compound. An exception to this behavior was observed for compound ZZW-142, which was the sole compound for which the simulation results gave a more reliable prediction. The computational results and the ITC measurements both indicate that there was no additive effect on the binding affinity obtained by introducing two different chemical groups to the same scaffold. In fact, the introduction of a methylene group slightly improved the binding, in the presence of the amine group in the alkyl tail (as visible in the comparison between the parent compound ZZW-115 and the derived analog ZZW-142); whereas the same modification strongly decreased the binding affinity of compound ZZW-130, which is the one with the best binding affinity for NUPR1 found in the experiment (leading to compound ZZW-144, which in contrast has a poor affinity). Although the non-additivity of molecular interactions is a well-known phenomenon that is recognized to hamper even the structure-based drug design of ligands targeting well-structured binding sites [44], this is likely to pose even greater difficulties in the case of ligand-based approaches against IDPs.

Another finding in our drug design effort was that our biological results (Figure 4, Figure 5 and Figure 6) indicated that a larger affinity, as measured by ITC, does not necessarily translate into a more favorable inhibitory activity. In fact, the results obtained in cellulo clearly indicated no clear correlation between ITC data and specific improvement in either of the following aspects: (i) better hampering of nuclear translocation of NUPR1; (ii) superior sensitizing of cells against DNA damage mediated by NUPR1; and (iii) more evident anticancer cell activity. In particular, the results observed as regards these three biological indicators were the poorest for ZZW-132 and ZZW-142, although such compounds are among the ones with the most favorable binding affinity for NUPR1 observed from ITC measurements (Table 1). This finding supports the notion that identifying the relationship between the chemical structure of compounds and their resulting biological activity, which is very difficult in the case of well-folded structures, is even more arduous for IDPs. At the moment, it is not clear how to further modify ZZW-115 to obtain better biological effects, although a number of examples reported in the literature of drug design against IDPs are encouraging in this sense [45,46].

Although it is difficult to identify the origin of the lack of correlation between our biophysical and biological results, it is interesting to suggest at least some possible explanations. First, NUPR1 is an IDP with a totally unfolded structure and it remains completely disordered to carry out its different functions, even in the presence of its natural partners (such as DNA or other proteins [12,15]) or when bound to TFP or TFP-derived drugs [25,26]. A compound binding with a high affinity may freeze the inherent internal flexibility of NUPR1, impacting its activity in other ways. It is worth to note that a residual flexibility has been shown to be necessary to carry out the function of a number of proteins: for instance, neurotensin needs to maintain its flexibility around the crucial residue Tyr11 when the protein is bound to neurotensin receptor 1 [47], and the same applies for a whole nascent helix of dynein when it is bound to dynactin [48]. As a second possible explanation for the biological effects we observed, it has been recently argued that an effective strategy for drug discovery in IDPs relies in exploiting the entropic contributions due to the protein chain flexibility, which would increase the affinity towards ligands by shifting the population of disordered conformations [49,50]. This approach (to increase disorder-upon-binding by shifting the fraction of disordered conformations of an IDP) has been successfully used in some cases, such as in the design of a drug targeting the kinase activation loop of Bcr-Abl [51], and can even work for well-folded proteins, as it helped in the identification of carbohydrate ligands targeting galectin-3 [52]; furthermore, such approach was instrumental for the entropy gain related to the lower susceptibility to resistance-associated mutations of certain inhibitors of the HIV-1 protease [53]. In the case of NUPR1, compounds ZZW-132 and ZZW-142 may shift the conformational populations of NUPR1 by increasing its flexibility, but such a shift could reduce the fraction of conformers that are active to perform some tasks (for instance, to allow NUPR1 translocation through the nuclear pore). Whether the explanation is either of the two proposed here or a different one, such effects are likely to have a role in the drug design against IDPs in general, and not only for NUPR1. These concerns supplement those that already exist in any drug development attempt, including those against well-folded protein targets, such as the fact that differences might also be due to other causes that include: (i) variations in the cellular uptake of the modified compounds; (ii) alterations in their cellular metabolism; (iii) the relative favorable binding affinity of the designed compounds for other proteins or biological substrates [54]; or even (iv) the formation of aggregates (oligomeric species of NUPR1, in this case) triggered by the presence of the bound compounds. Thus, the biological effect of a given compound (primary effects plus side-effects) will depend on the binding affinity towards the primary target, the effective concentration of the compound at the appropriate target location, and the binding affinities towards unwanted targets.

## 5. Conclusions

In this work we have pursued the design of novel inhibitors of the tumorigenic protein NUPR1, and suggested that a subtle compromise between improving ligand affinity and maintaining protein functionality may be required. Whereas increasing the affinity towards NUPR1 is important to obtain a more potent drug, the functional pathways this protein belongs to should not be exceedingly perturbed, in order to maintain their effectiveness in performing some of their biological roles. This poses significant limits in the possibility of relying solely on the most traditional theoretical modelling techniques, including molecular docking and MD. A counterintuitive consequence is that, for disordered proteins performing multiple functions within cells, an improved inhibitory effect may be attained by investigating in cellulo the properties of compounds with a comparable—or even slightly reduced—binding affinity with respect to a lead drug compound (such as ZZW-115, in our case). Therefore, we provide a direct evidence that also for IDPs the best drug is not necessarily the best binder. This finding enlarges the possibility in the number of active molecules that can be hoped to be effective against IDPs, encouraging the exploration of regions of the chemical space of drug compounds that may be currently neglected or underrated.

## Figures and Tables

**Table 1:** Thermodynamic parameters of the binding reaction between NUPR1 and the compounds. ^a^ Obtained from ITC measurements; errors are estimated from the fitting and standard thermodynamic relationships (*K*_d_ = 1/*K*_a_; Δ*G* = −R*T* ln *K*_a_; Δ*G* = Δ*H* – *T*Δ*S*). ^b^ Data taken from [26].

Compound	*K*_a_ (M^–1^) × 10^5^	*K*_d_ (µM) (=1/*K*_a_) ^a^	Δ*G* (kcal mol^–1^) ^a^	Δ*H * (kcal mol^–1^) ^a^	−*T*Δ*S * (kcal mol^–1^) ^a^	N ^a^
ZZW-129	3.1 ± 0.3	3.2 ± 0.3	−7.5 ± 0.1	−0.7 ± 0.4	−6.8 ± 0.4	1.2 ± 0.1
ZZW-130	6.4 ± 0.4	1.6 ± 0.1	−7.9 ± 0.1	0.3 ± 0.3	−8.2 ± 0.3	1.4 ± 0.1
ZZW-131	4.7 ± 0.3	2.1 ± 0.1	−7.7 ± 0.1	0.5 ± 0.3	−8.2 ± 0.3	0.8 ± 0.1
ZZW-132	5.8 ± 0.4	1.7 ± 0.1	−7.9 ± 0.1	0.3 ± 0.3	−8.2 ± 0.3	1.2 ± 0.1
ZZW-142	4.9 ± 0.3	2.0 ± 0.2	−7.8 ± 0.1	0.3 ± 0.3	−8.1 ± 0.3	1.3 ± 0.1
ZZW-143	0.5 ± 0.1	20 ± 4	−6.4 ± 0.1	0.8 ± 0.4	−7.2 ± 0.4	1.2 ± 0.1
ZZW-144	0.85 ± 0.8	12 ± 1	−6.7 ± 0.1	0.5 ± 0.5	−7.2 ± 0.5	1.2 ± 0.1
ZZW-145	0.82 ± 0.9	11 ± 1	−6.7 ± 0.1	0.7 ± 0.5	−7.4 ± 0.5	1.2 ± 0.1
ZZW-148	1.0 ± 0.1	9.6 ± 1.0	−6.8 ± 0.1	0.7 ± 0.5	−7.5 ± 0.5	1.2 ± 0.1
ZZW-115 ^b^	4.7 ± 0.4	2.1 ± 0.2	−7.7 ± 0.1	−0.4 ± 0.3	−7.3 ± 0.3	0.9 ± 0.1
TFP ^b^	1.9 ± 0.2	5.2 ± 0.6	−7.2 ± 0.1	−1.1 ± 0.4	−6.1 ± 0.4	1.0 ± 0.1

**Table 2:** IC_50_ (in µM) for the different assayed cell-lines with the ZZW-115-derived compounds.

	PDAC001T	PDAC012T	PDAC021T	PDAC081T	PDAC082T	PDAC087T	PDAC088T	PDAC089T	PDAC115T	MiaPaCa-2
ZZW-129	1.94	2.77	3.41	0.86	3.56	4.28	7.28	7.90	2.46	0.33
ZZW-130	2.45	4.18	3.81	1.12	4.33	4.95	6.75	6.03	2.91	0.35
ZZW-131	3.98	5.25	6.47	0.97	6.49	4.43	8.11	10.59	5.75	0.30
ZZW-132	12.47	12.21	16.50	1.91	21.10	16.55	23.63	29.10	16.06	1.04
ZZW-142	11.93	12.78	17.01	2.27	22.97	16.55	23.92	22.46	14.70	0.95
ZZW-143	2.31	2.18	2.57	0.97	3.55	2.22	5.94	6.12	2.50	0.29
ZZW-144	2.51	2.51	3.69	0.98	3.04	3.57	5.80	8.38	3.22	0.26
ZZW-145	2.36	2.36	2.33	0.78	2.33	2.37	5.00	6.22	2.44	0.19
ZZW-148	3.75	3.70	4.35	1.11	4.10	6.57	10.73	14.77	4.37	0.47
ZZW-115	1.84	2.44	2.54	0.84	2.36	3.72	5.29	4.72	2.21	0.32
TFP	12.48	20.39	23.51	2.48	21.47	7.80	15.90	8.09	10.12	9.28

## Data Availability

Materials are available from any of the corresponding authors upon reasonable request.

## Supplementary Materials

The following are available online at https://www.mdpi.com/article/10.3390/biom11101453/s1. Scheme S1: Synthesis of ZZW-115-derived compounds. Figure S1: capacity of ZZW-115-derived compounds to perform nuclear translocation. Figure S2: capacity of ZZW-115-derived compounds to sensitize 5-FU induced DNA damage. The ^1^H, ^13^C-NMR spectra and HPLC spectra of the compounds are also included.
