# Supplementary material for: Design of Inhibitors of the Intrinsically Disordered Protein NUPR1: Balance between Drug Affinity and Target Function
Source: Biomolecules. 2021 Oct 3;11(10):1453. doi: 10.3390/biom11101453 (PMC8533202; doi:10.3390/biom11101453)
Supplement: Supplementary file 1 [file biomolecules-11-01453-s001.zip › biomolecules-1376295-supplementary.pdf]

Supplementary Information

Design of inhibitors of the intrinsically disordered protein NUPR1: balance between drug affinity and target function

Bruno Rizzuti, Wenjun Lan, Patricia Santofimia-Castaño, Zhengwei Zhou, Adrián Velázquez-Campoy, Olga Abián, Ling Peng, José L. Neira, Yi Xia and Juan L. Iovanna

Table of Contents

• Scheme S1: Synthesis of ZZW-115-derived compounds.

• Fig. S1: capacity of ZZW-115-derived compounds to perform nuclear translocation

• Fig. S2: capacity of ZZW-115-derived compounds to sensitize 5-FU induced DNA damage

• ^{1}H, ^{13}C-NMR spectra of the compounds

• HPLC spectra of the compounds

Scheme S1. Synthesis of ZZW-115-derived compounds.

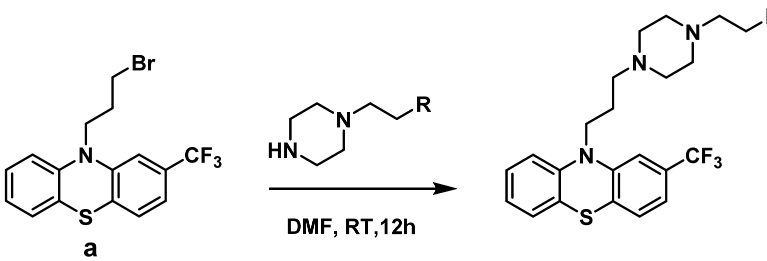

Name	R	Yield (%)	Name	R	Yield (%)
**ZZW-129**	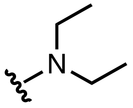	68	**ZZW-143**	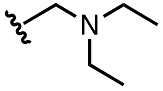	69
**ZZW-130**	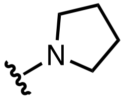	79	**ZZW-144**	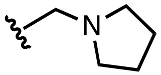	63
**ZZW-131**	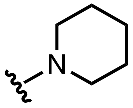	74	**ZZW-145**	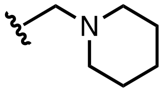	76
**ZZW-132**	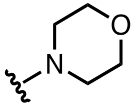	71	**ZZW-148**	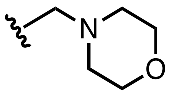	70
**ZZW-142**	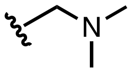	76			

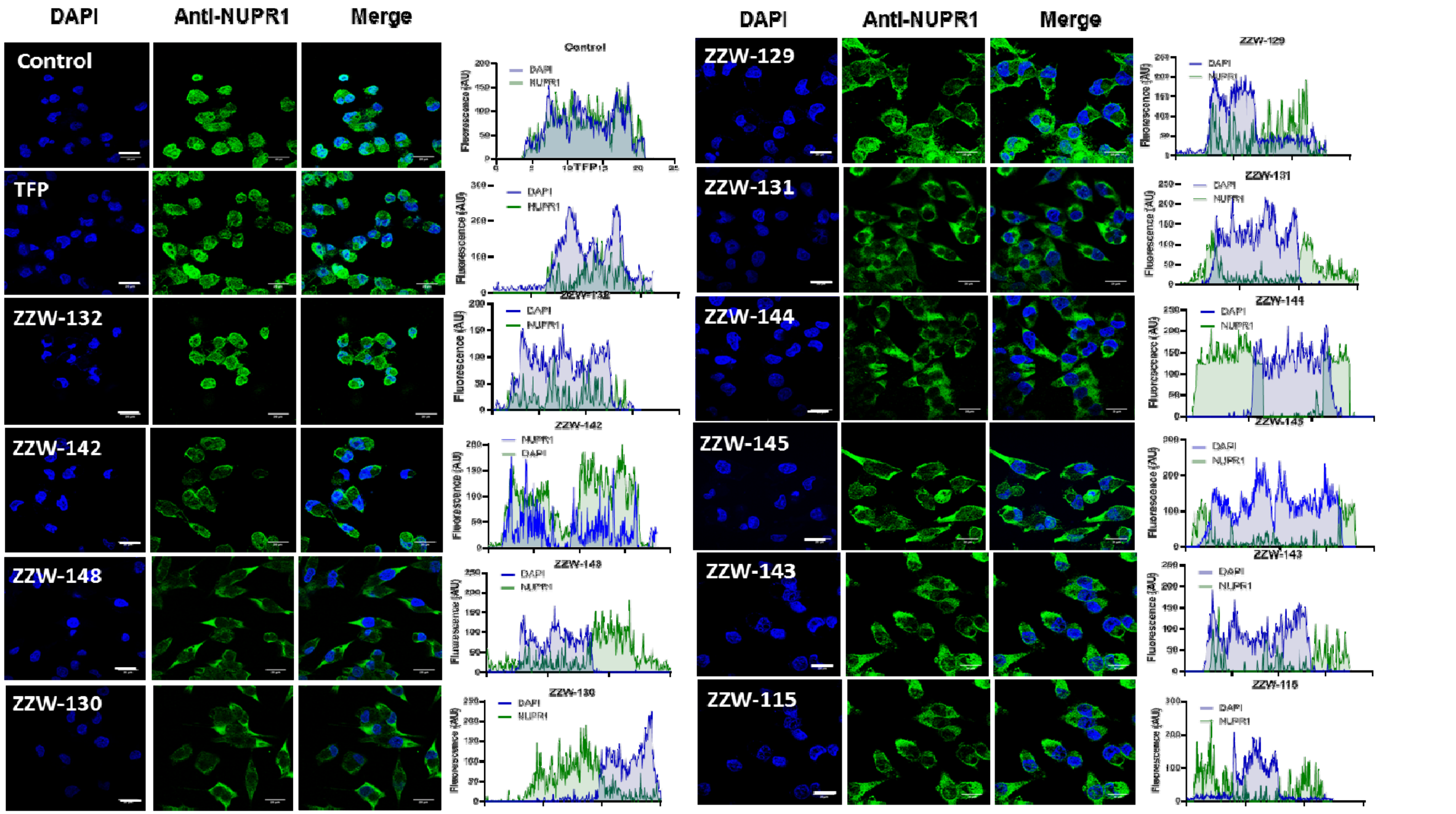

Figure S1: Colocalization scatter plots of NUPR1; Pearson's R value (PRV), and Mander's coefficient (MC) were calculated by using the ImageJ Coloc2 plugin. The bar indicates 20 μm.

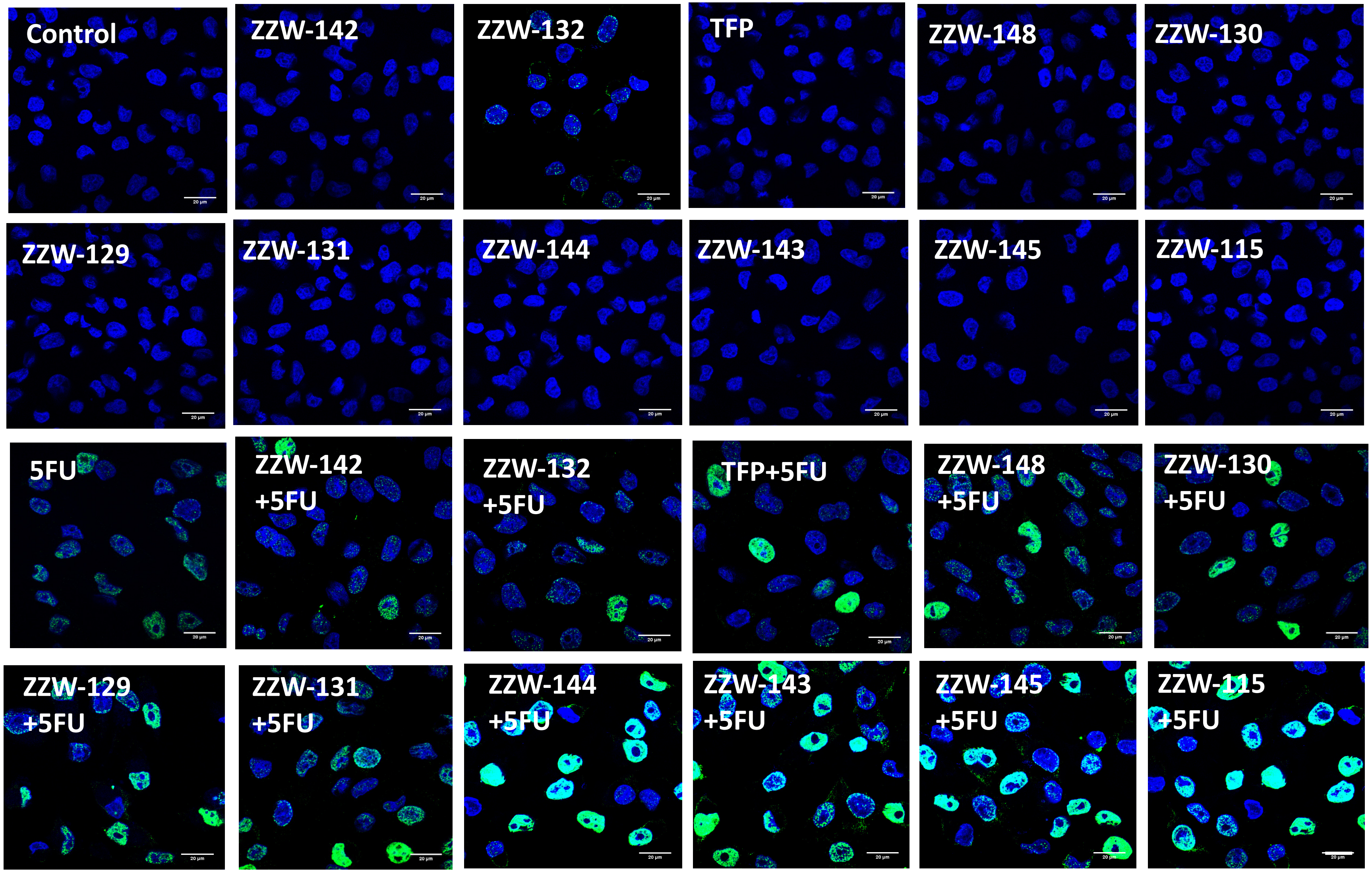

Figure S2: Intensity profiles shown for the effect of the ZZW-derived compounds for the ability to sensitize 5-FU induced damage. The bar indicates 20 μm.

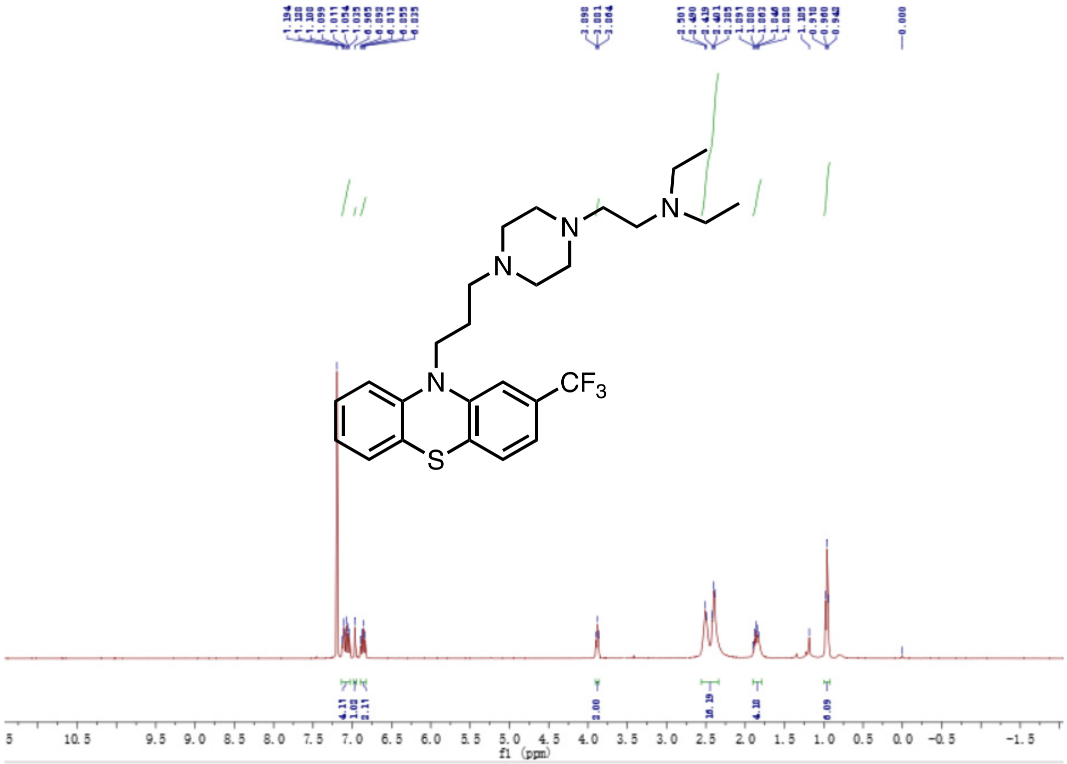

^{1}H-NMR of **ZZW-129**

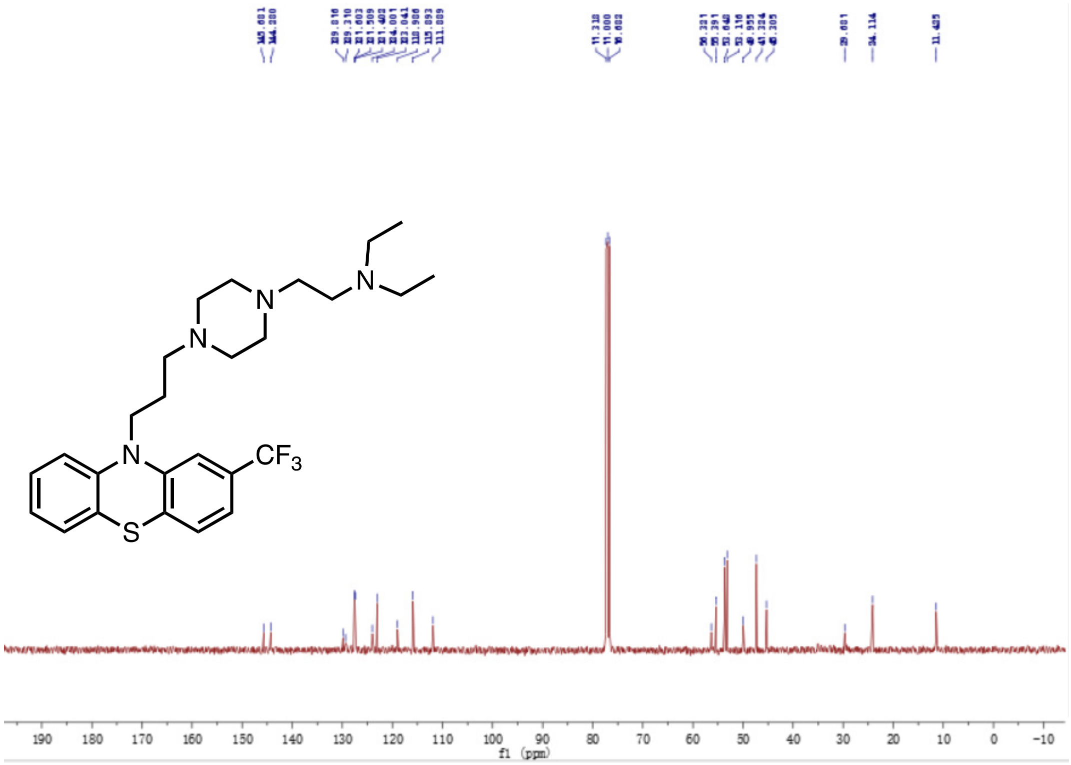

^{13}C-NMR of **ZZW-129**

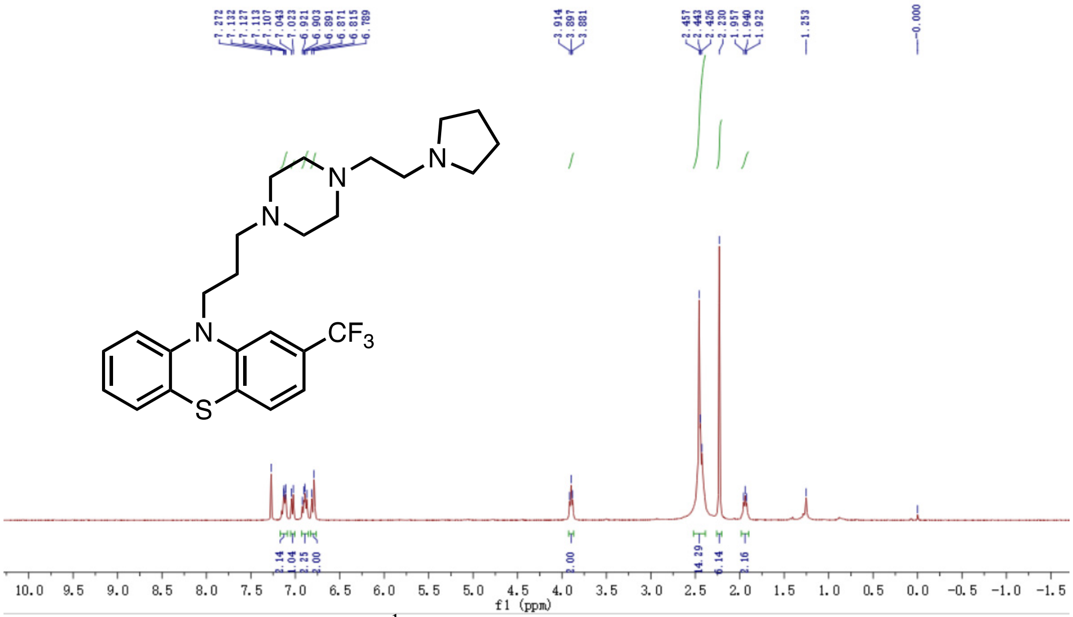

^{1}H-NMR of **ZZW-130**

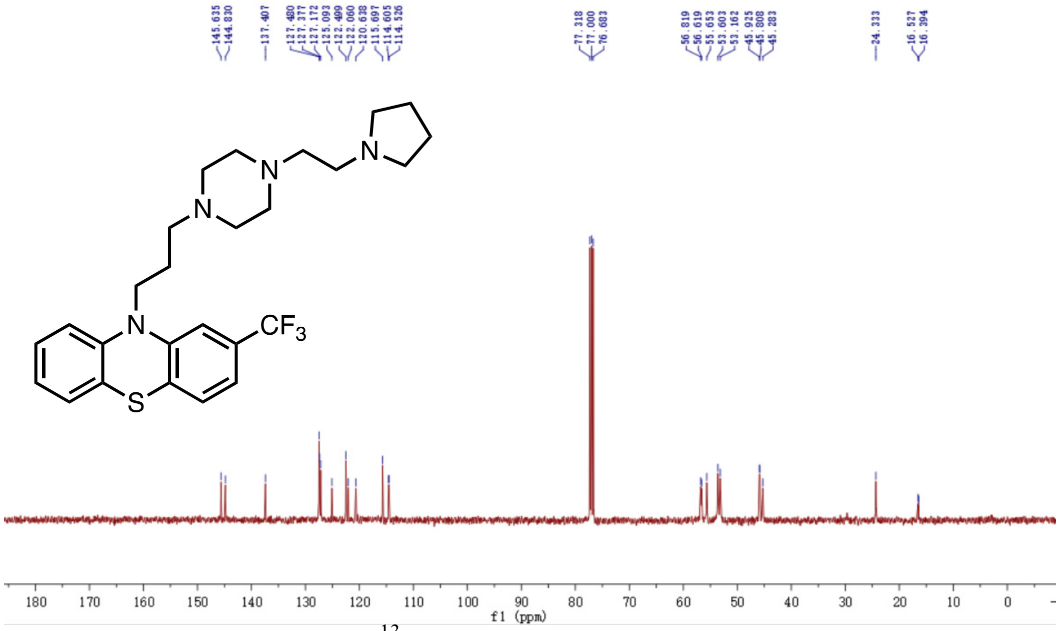

^{13}C-NMR of **ZZW-130**

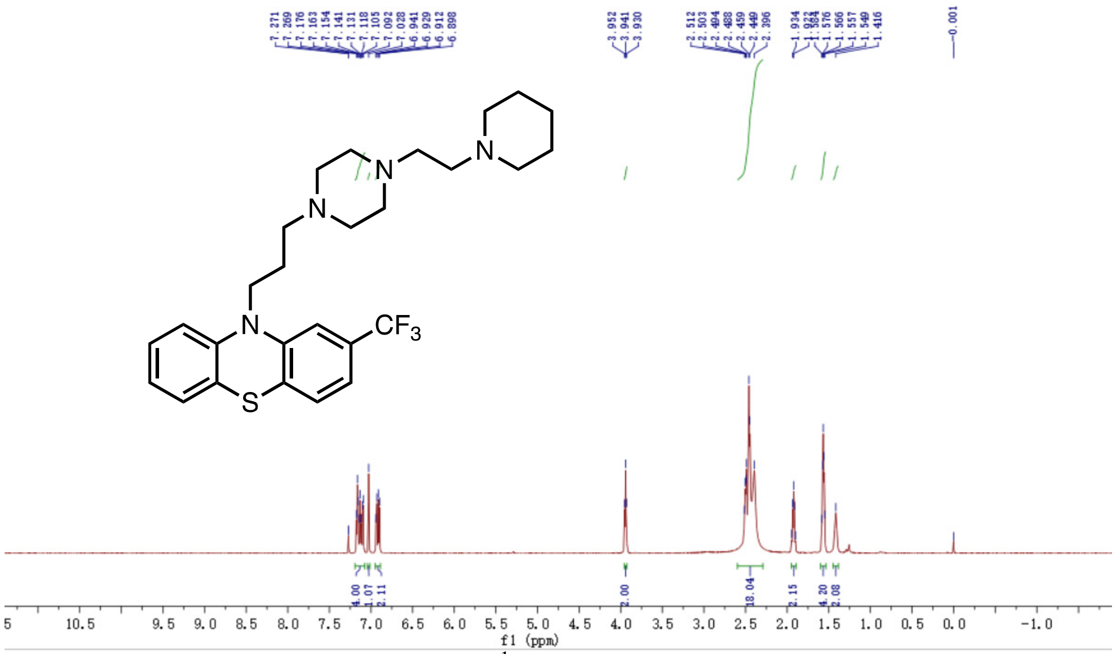

^{1}H-NMR of **ZZW-131**

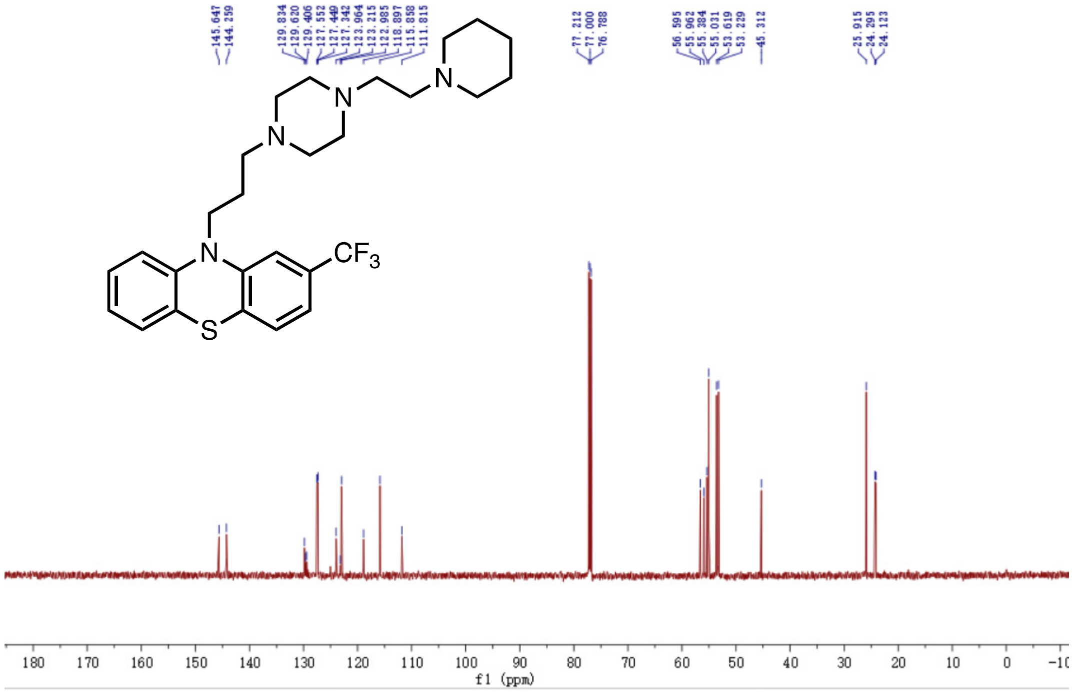

^{13}C-NMR of **ZZW-131**

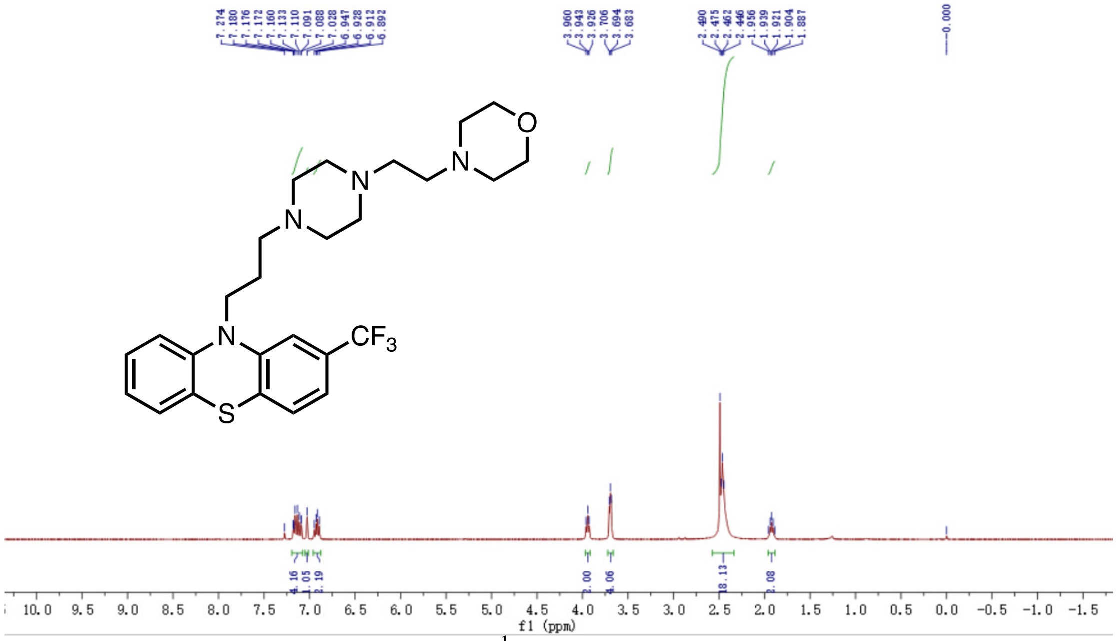

^{1}H-NMR of **ZZW-132**

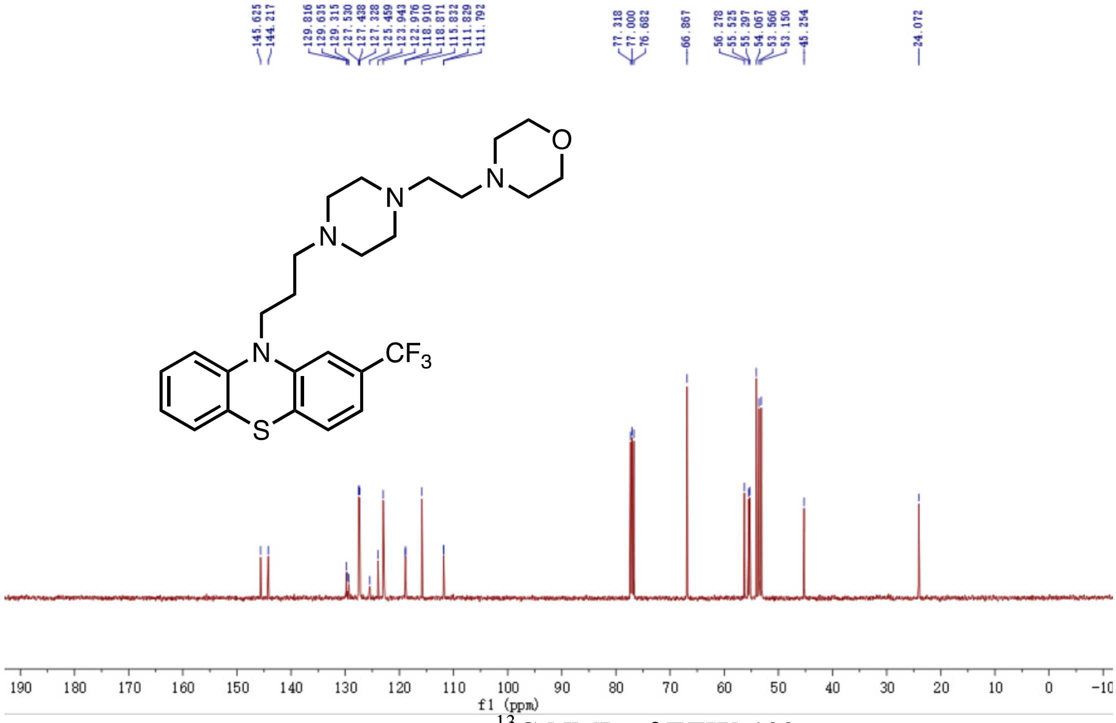

^{13}C-NMR of **ZZW-132**

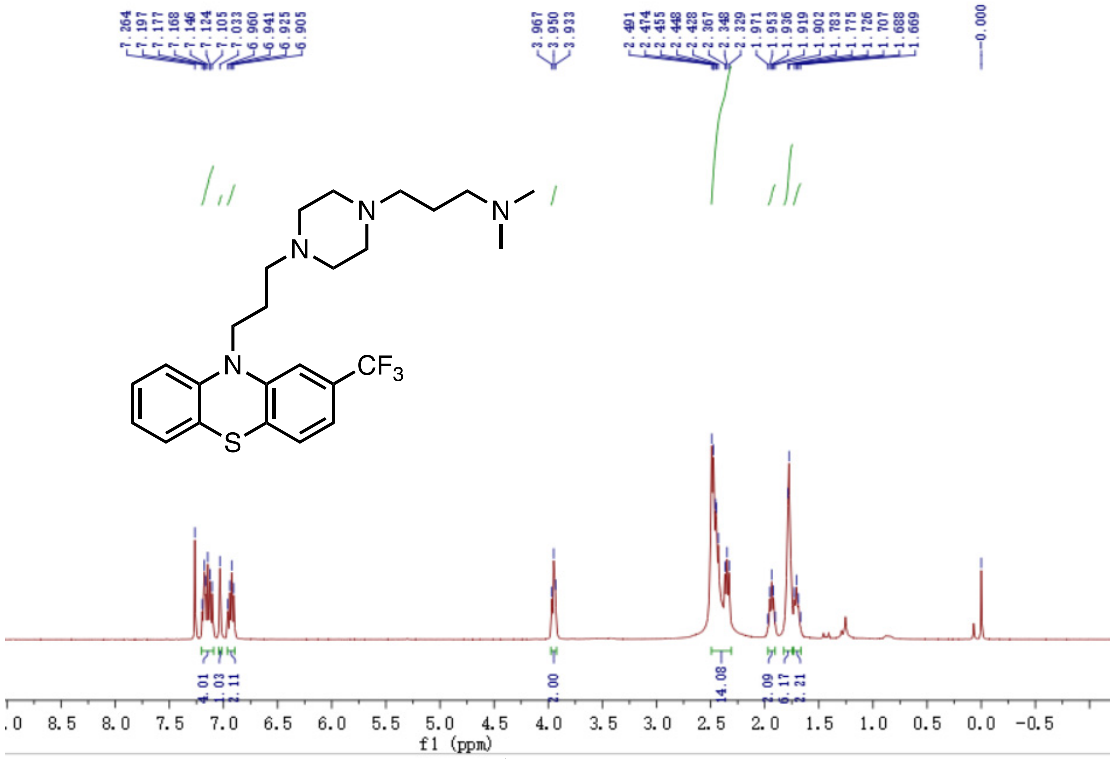

^{1}H-NMR of **ZZW-142**

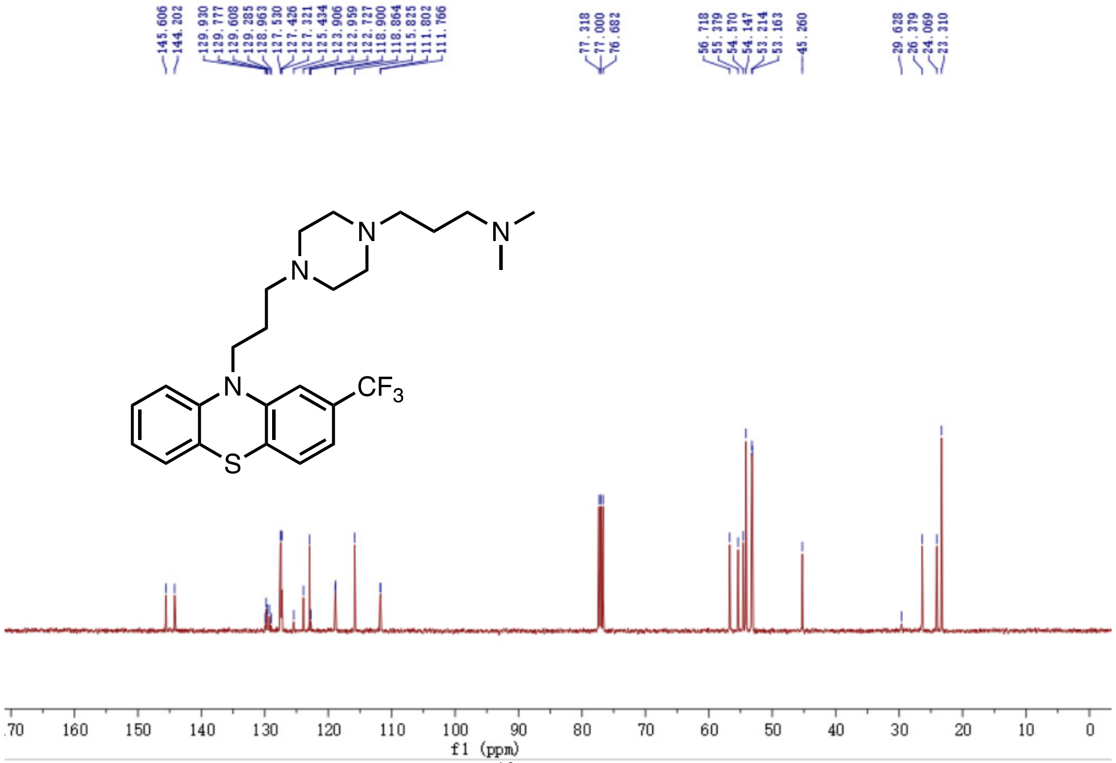

^{13}C-NMR of **ZZW-142**

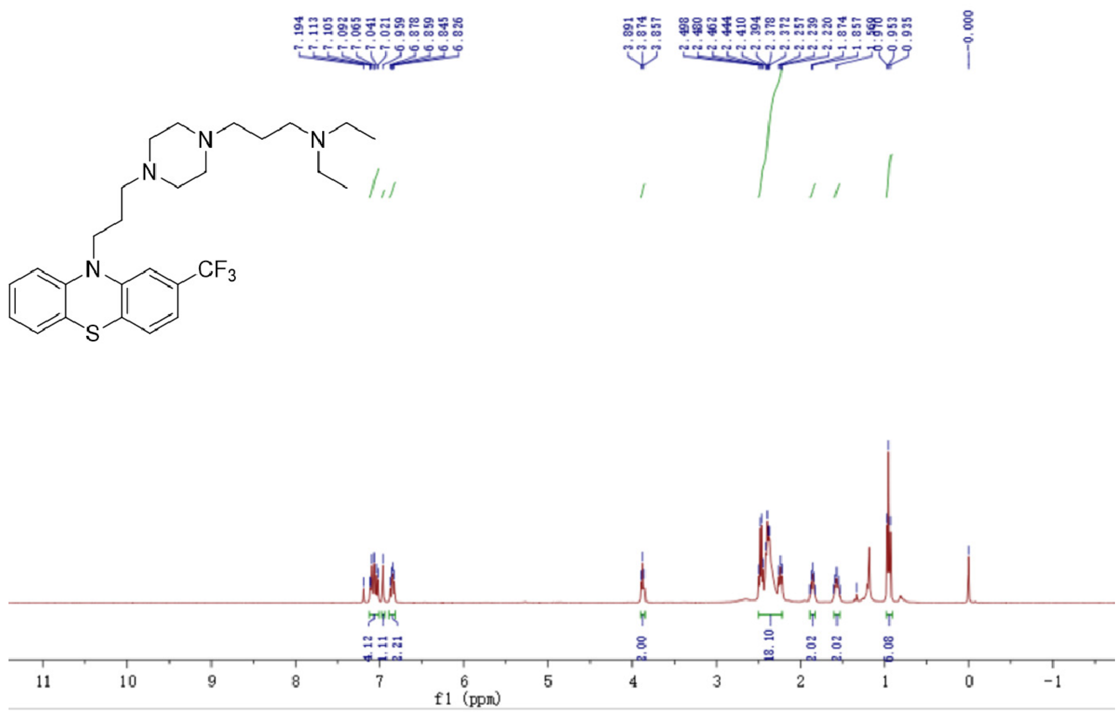

^{1}H-NMR of **ZZW-143**

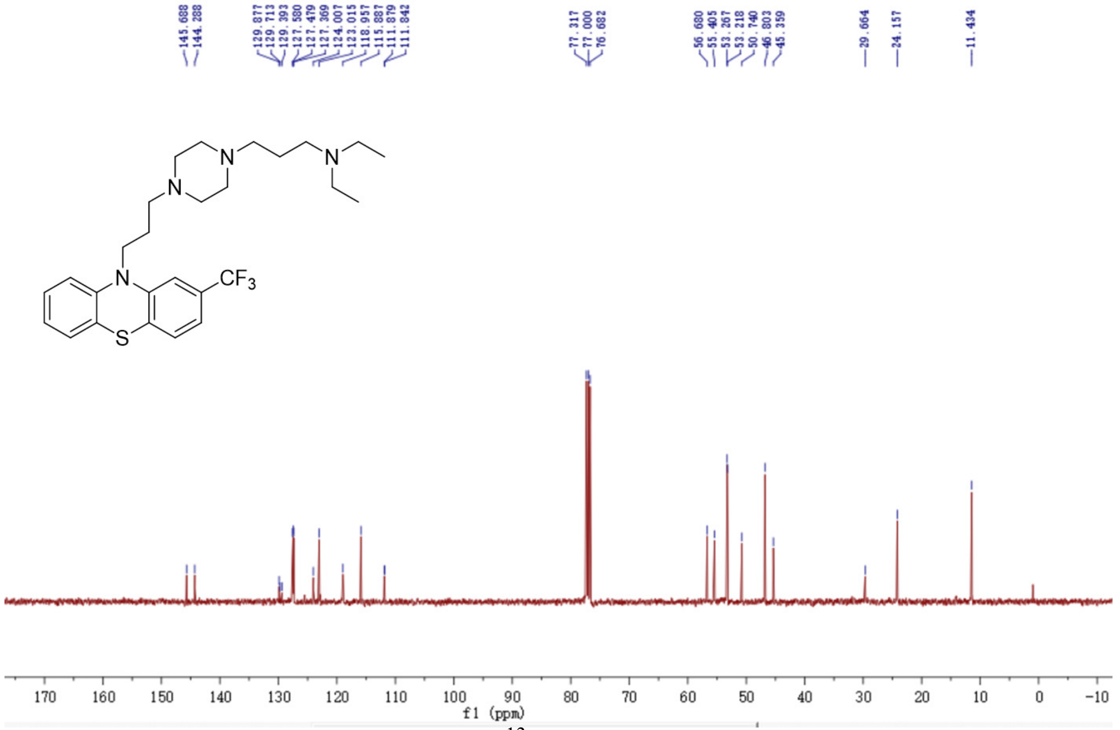

^{13}-NMR of **ZZW-143**

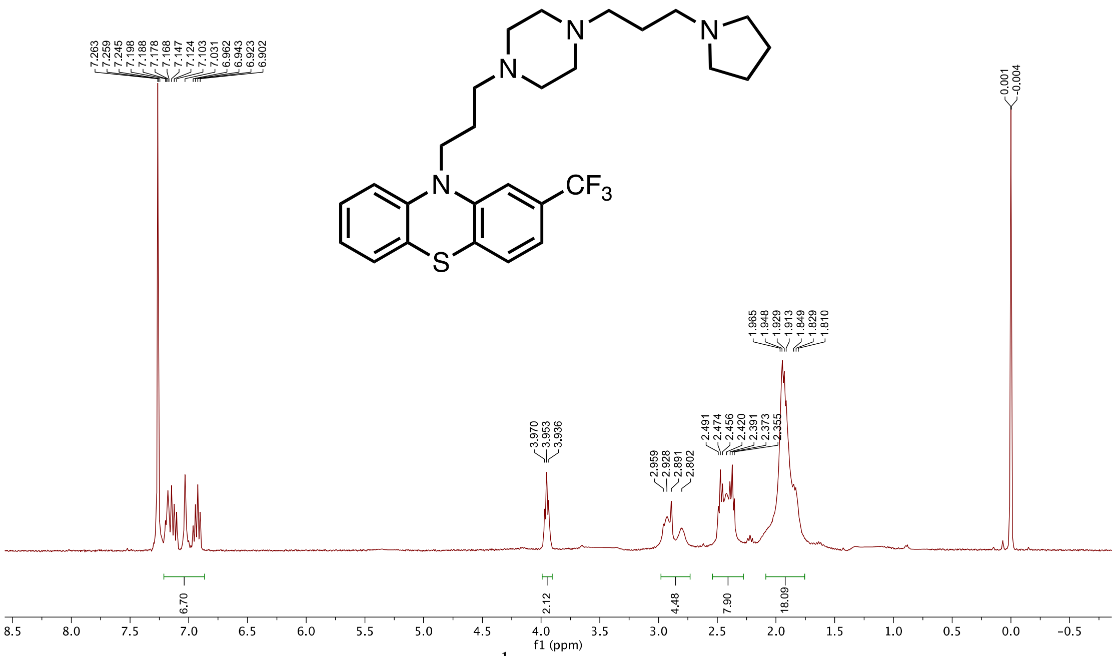

^{1}H-NMR of **ZZW-144**

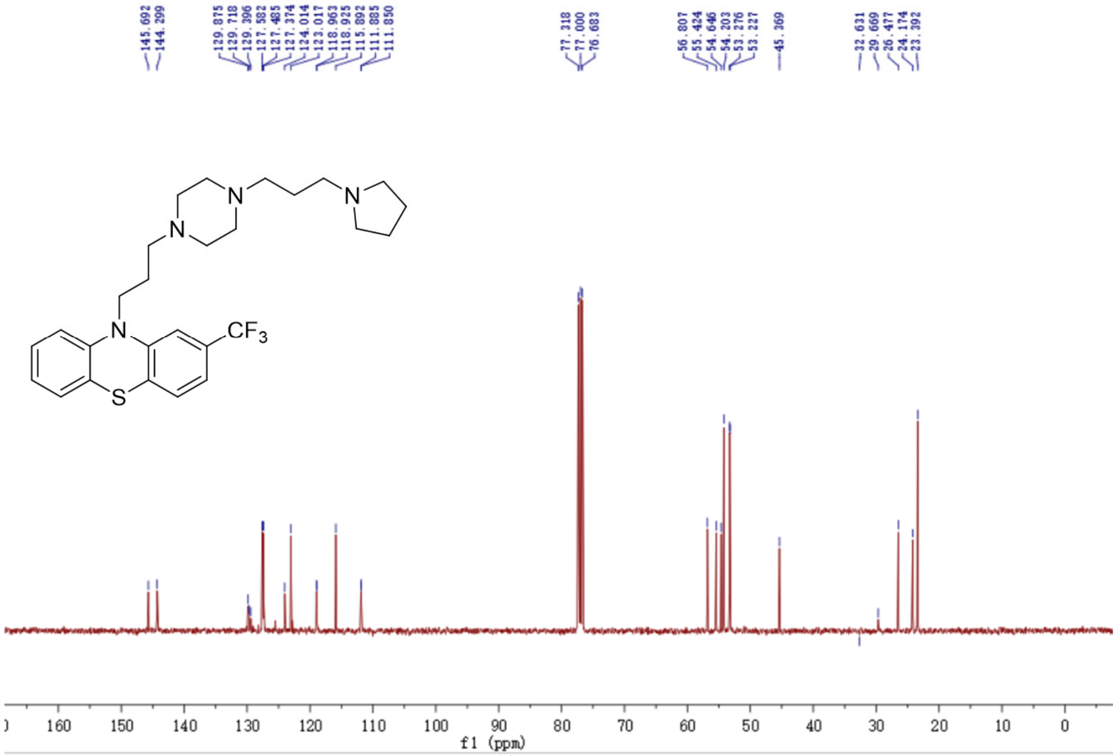

^{13}C-NMR of **ZZW-144**

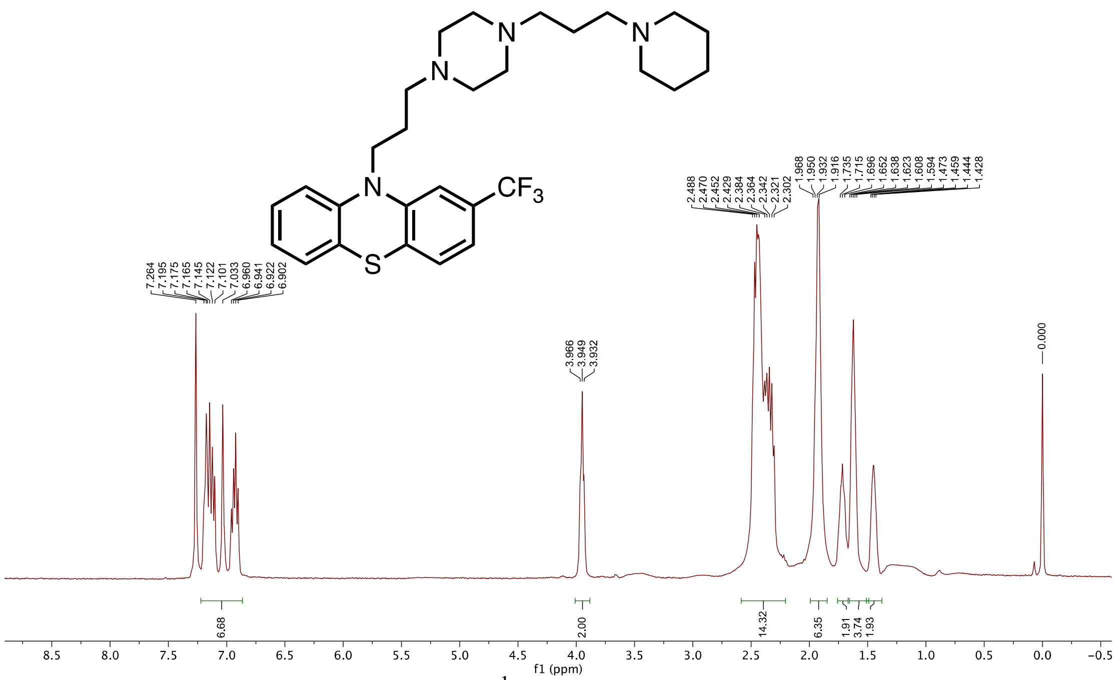

^{1}H-NMR of **ZZW-145**

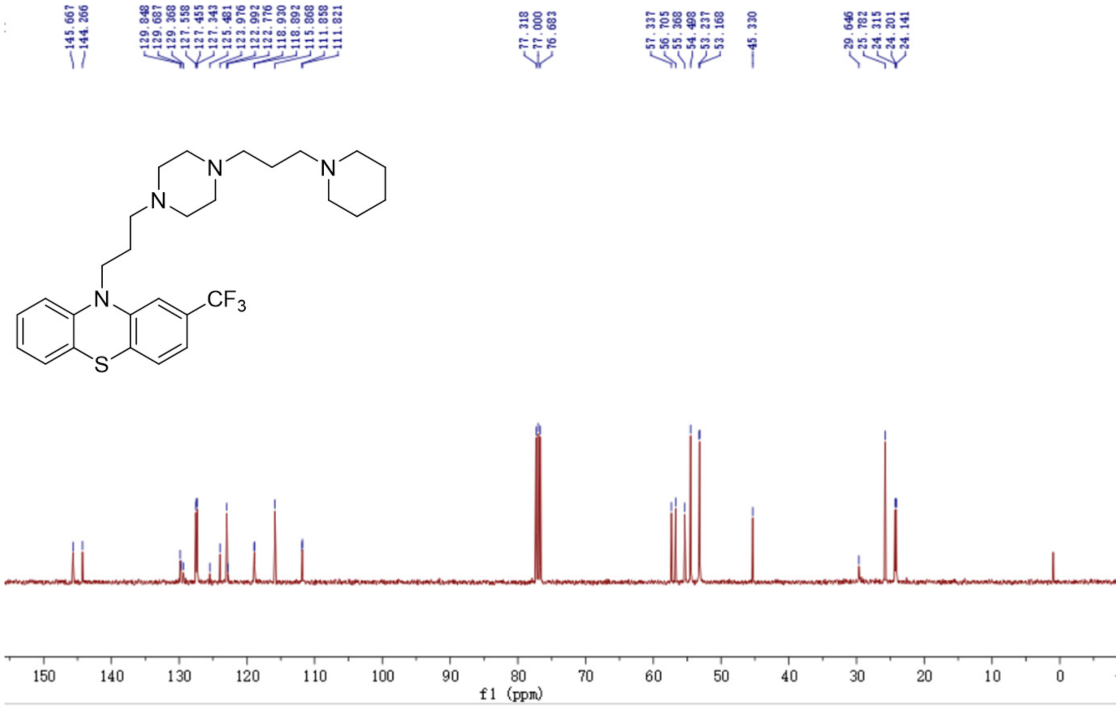

^{13}C-NMR of **ZZW-145**

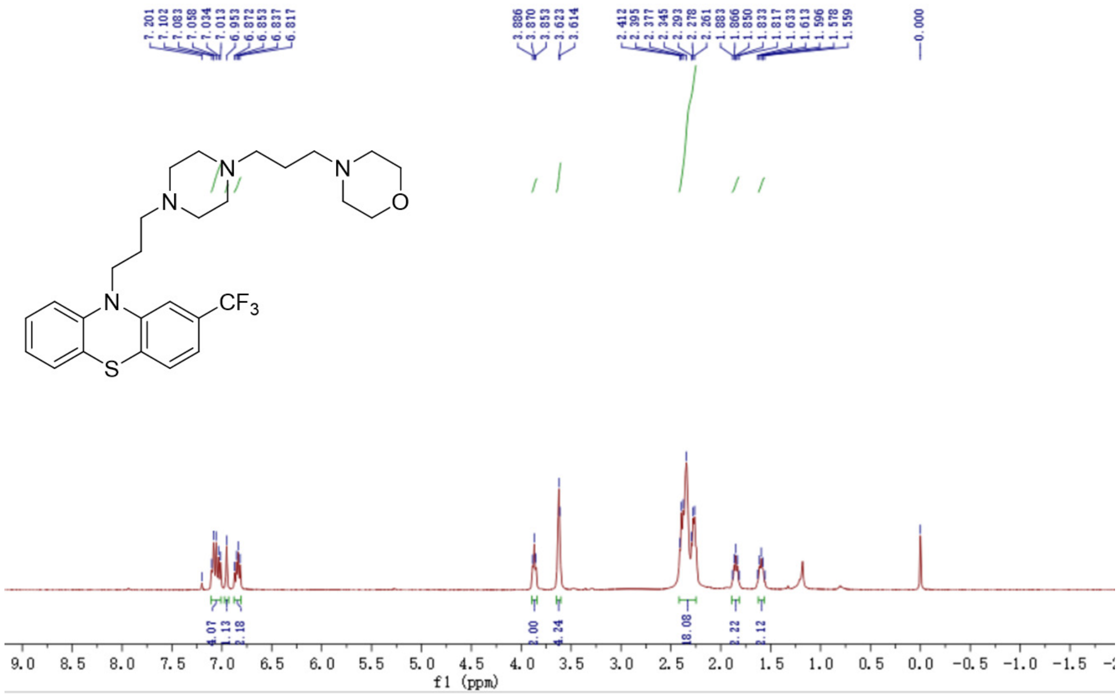

^{1}H-NMR of **ZZW-148**

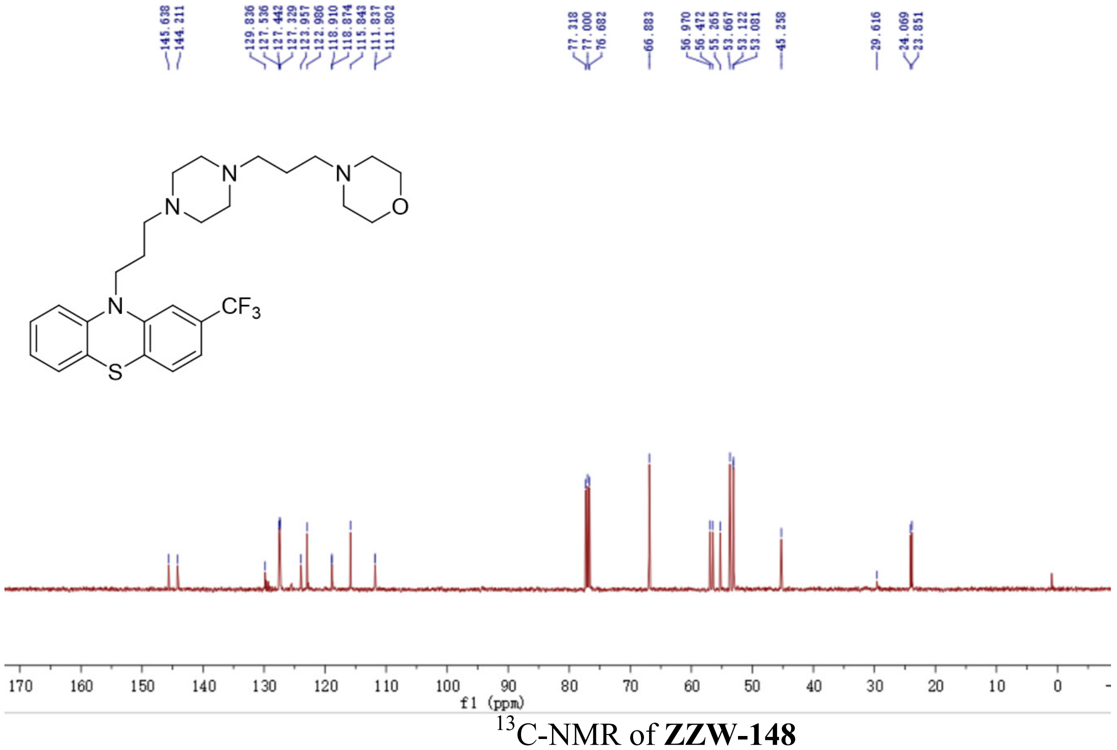

^{13}C-NMR of **ZZW-148**

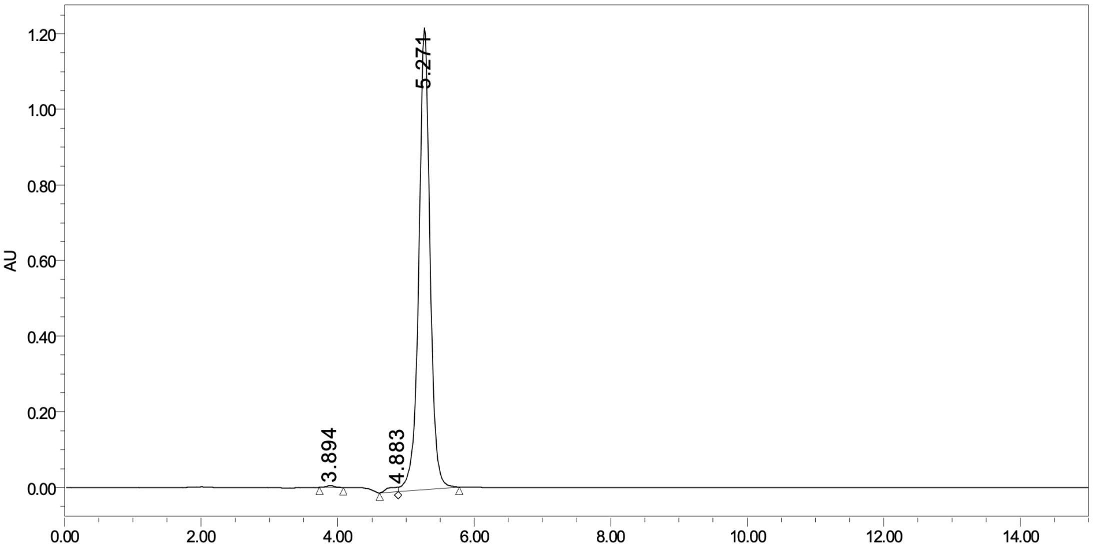

HPLC analysis of **ZZW-129** (Method 1)

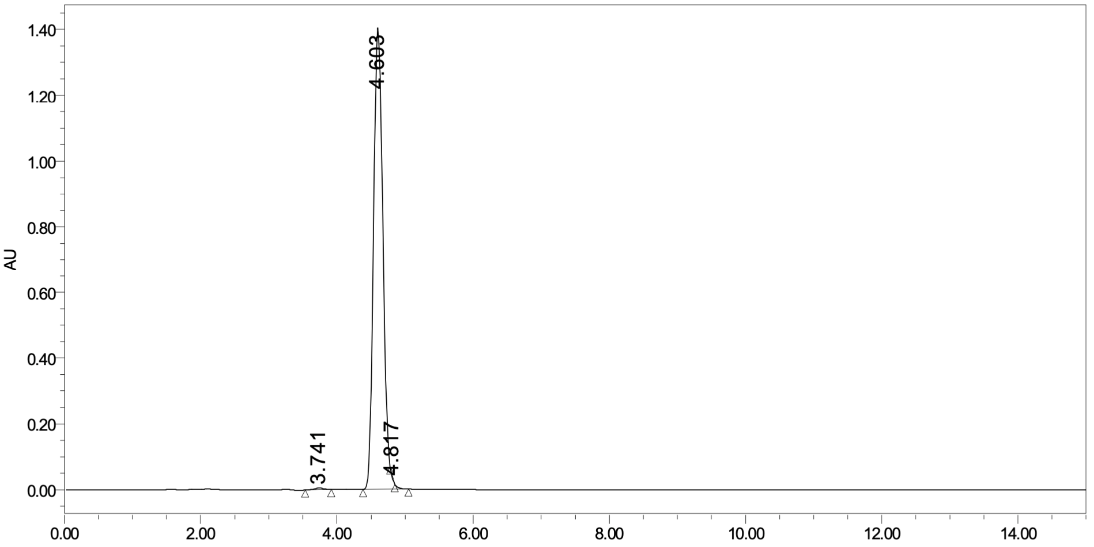

HPLC analysis of **ZZW-129** (Method 2)

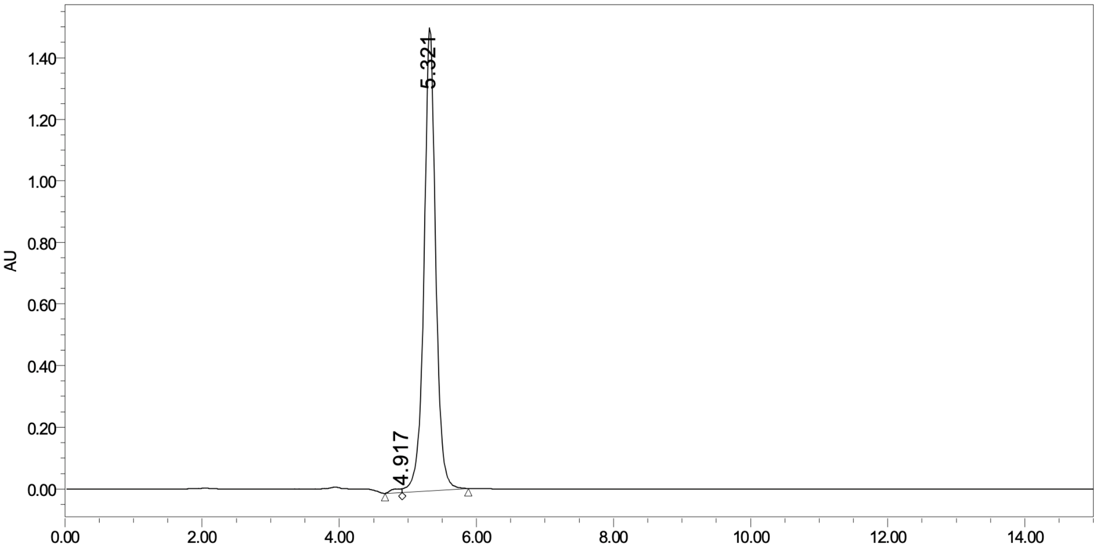

HPLC analysis of **ZZW-130** (Method 1)

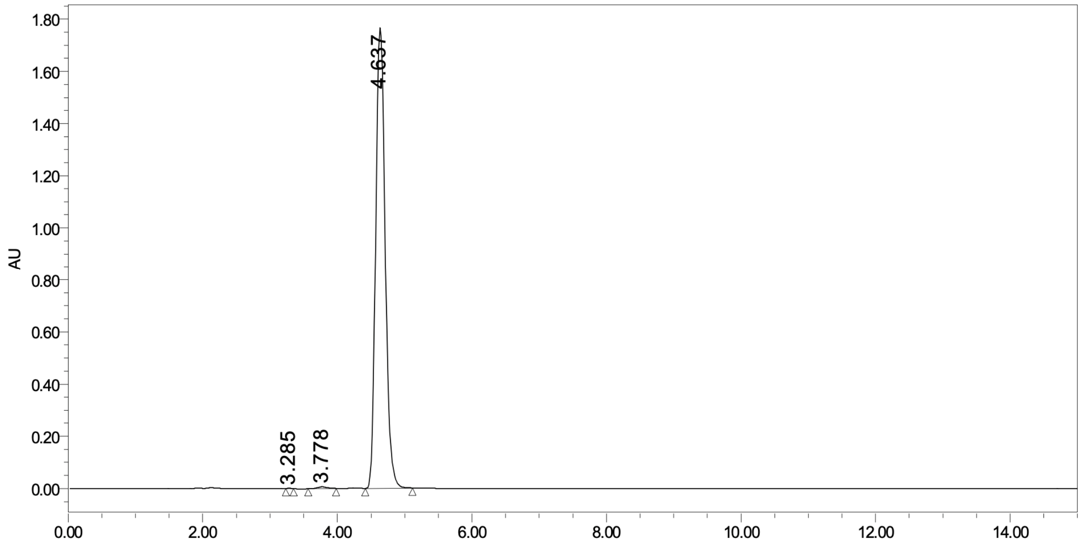

HPLC analysis of **ZZW-130** (Method 2)

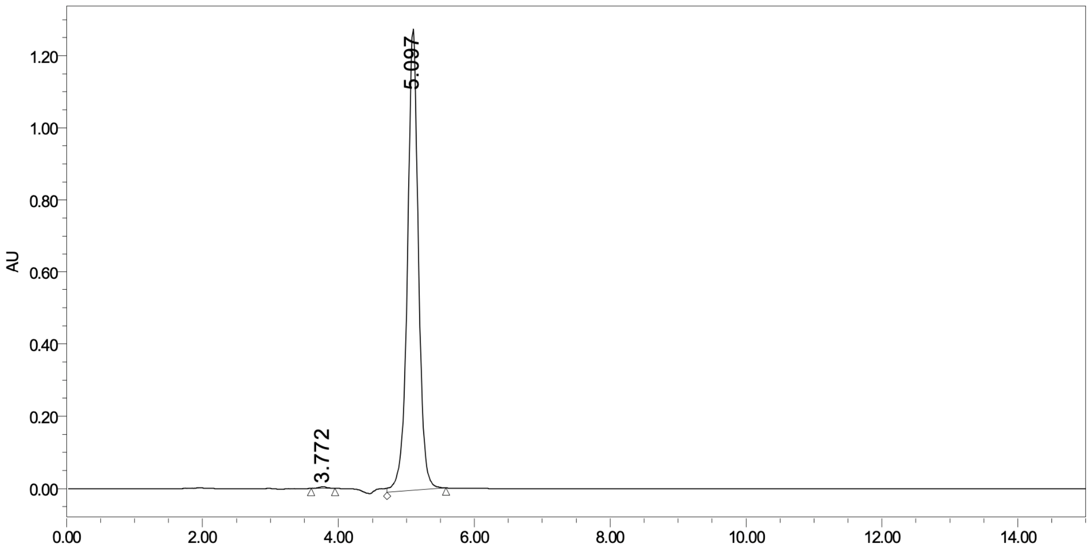

HPLC analysis of **ZZW-131** (Method 1)

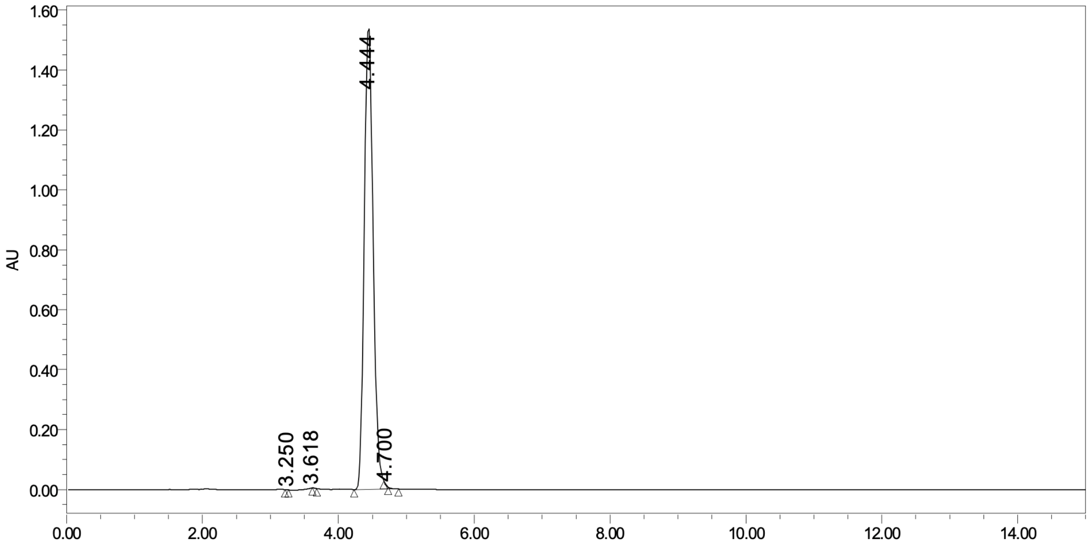

HPLC analysis of **ZZW-131** (Method 2)

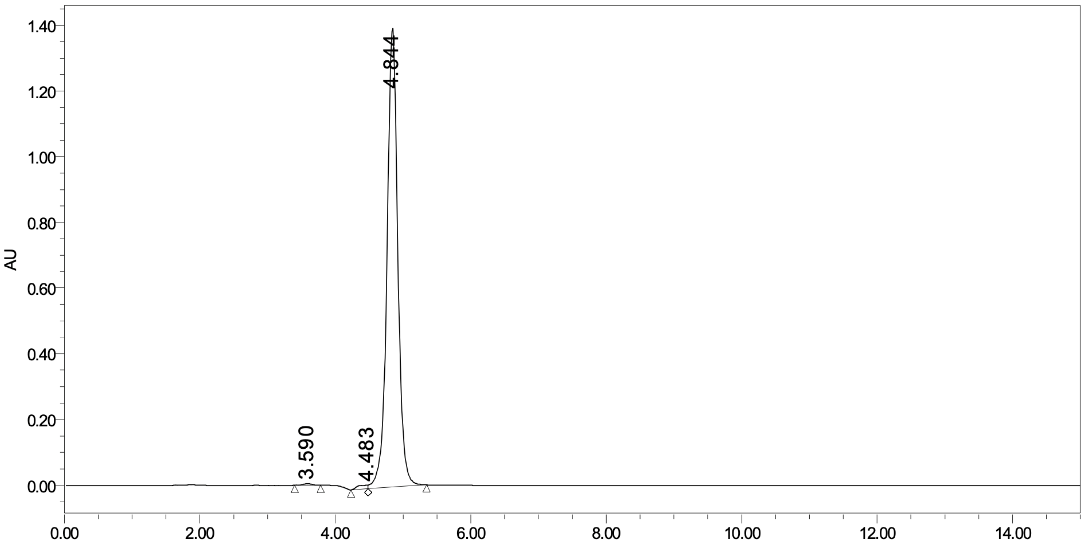

HPLC analysis of **ZZW-132** (Method 1)

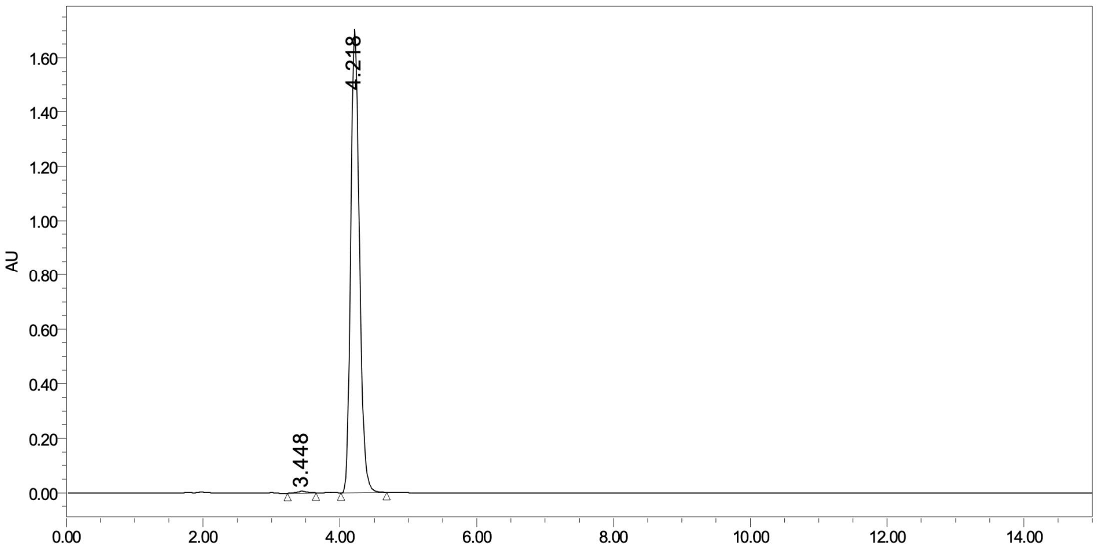

HPLC analysis of **ZZW-132** (Method 2)

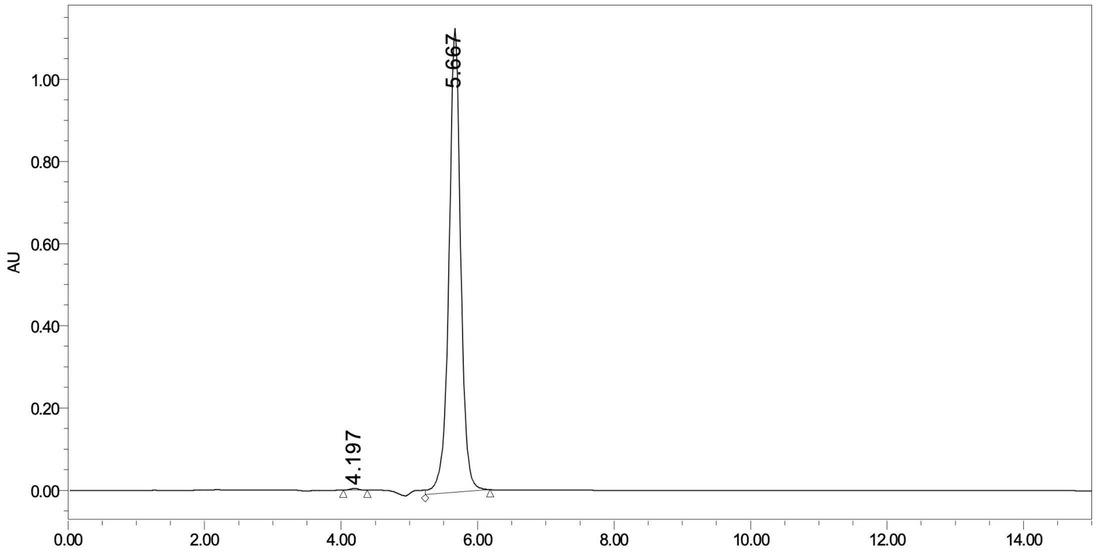

HPLC analysis of **ZZW-142** (Method 1)

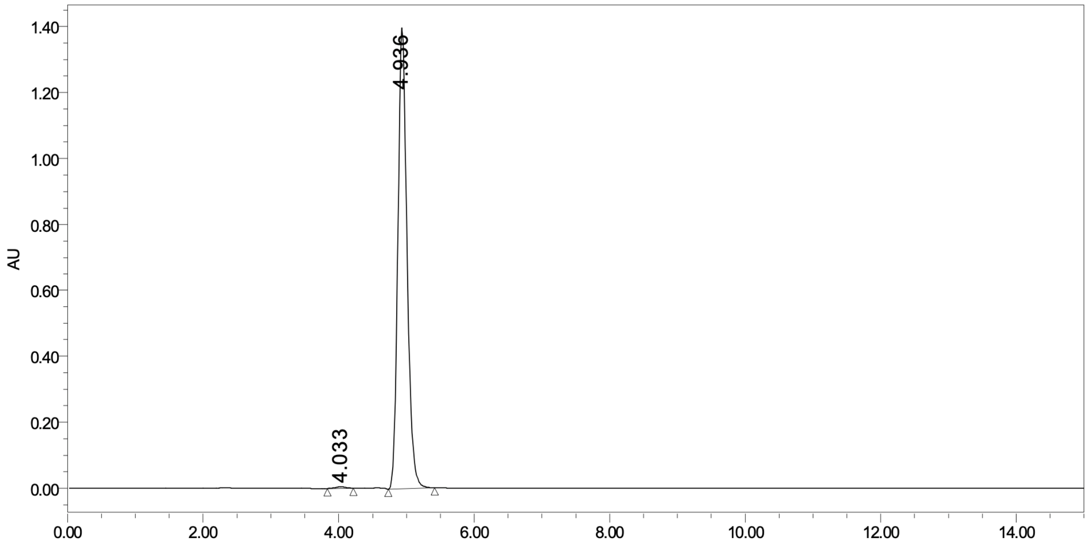

HPLC analysis of **ZZW-142** (Method 2)

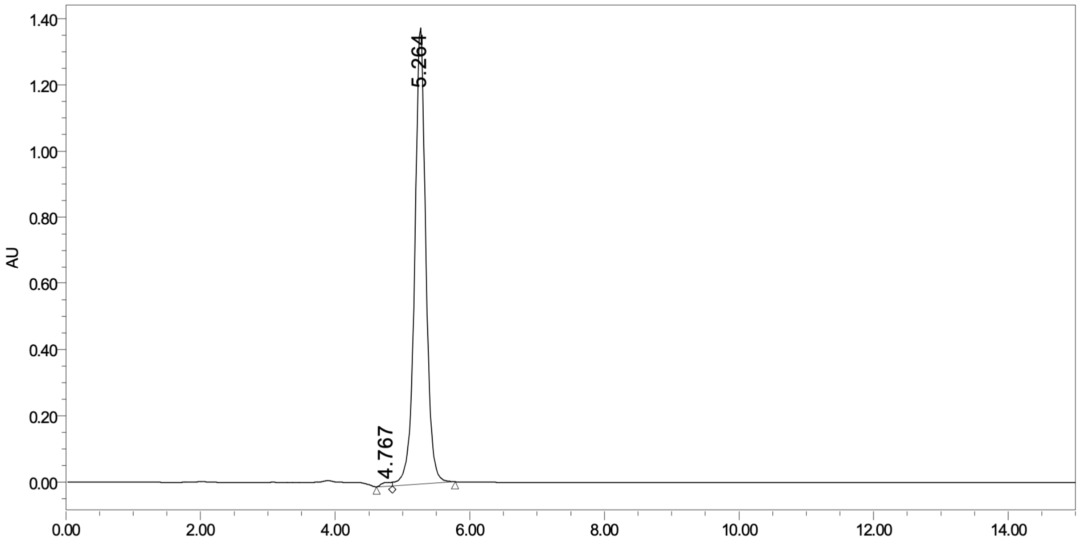

HPLC analysis of **ZZW-143** (Method 1)

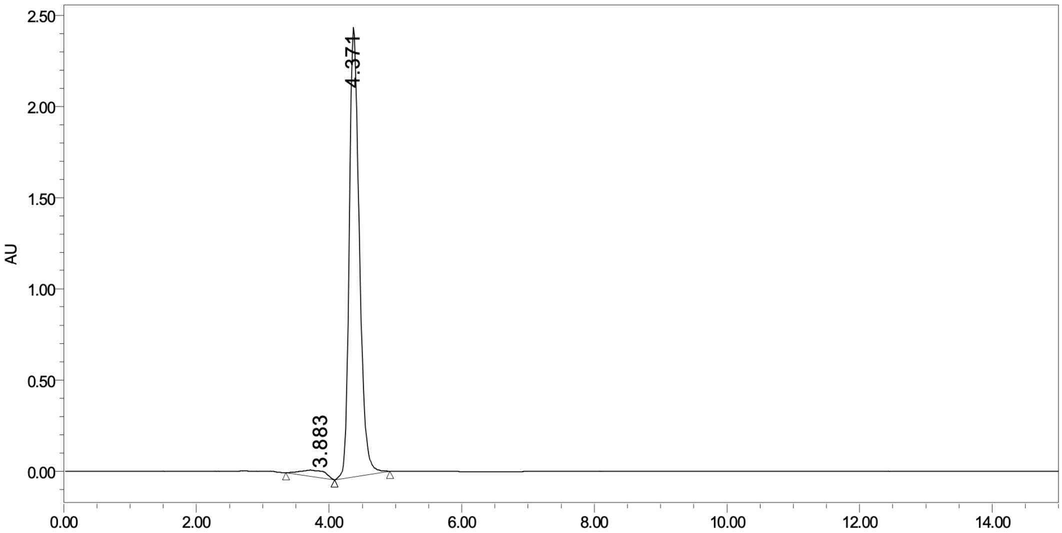

HPLC analysis of **ZZW-143** (Method 2)

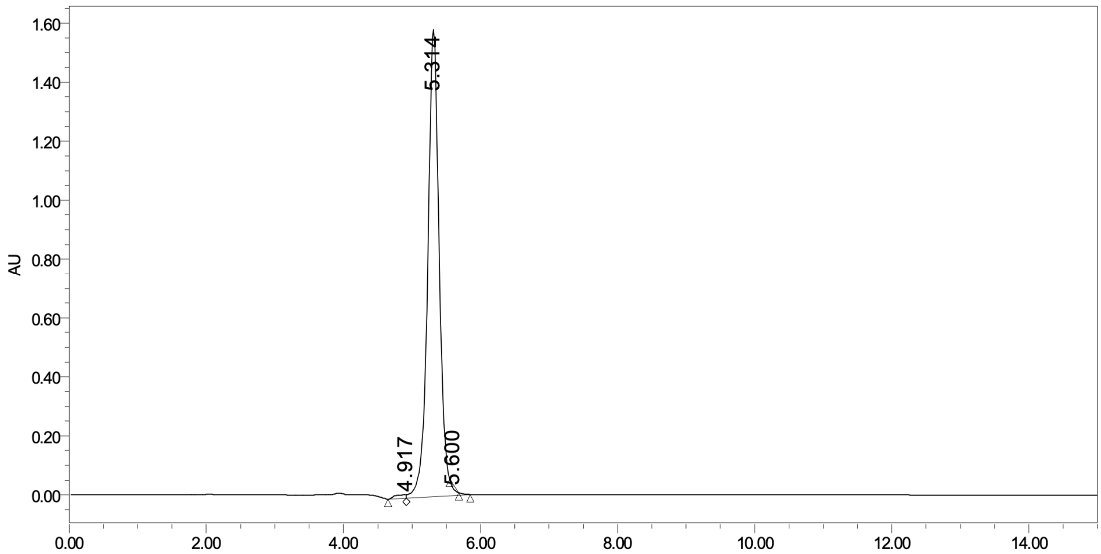

HPLC analysis of **ZZW-144** (Method 1)

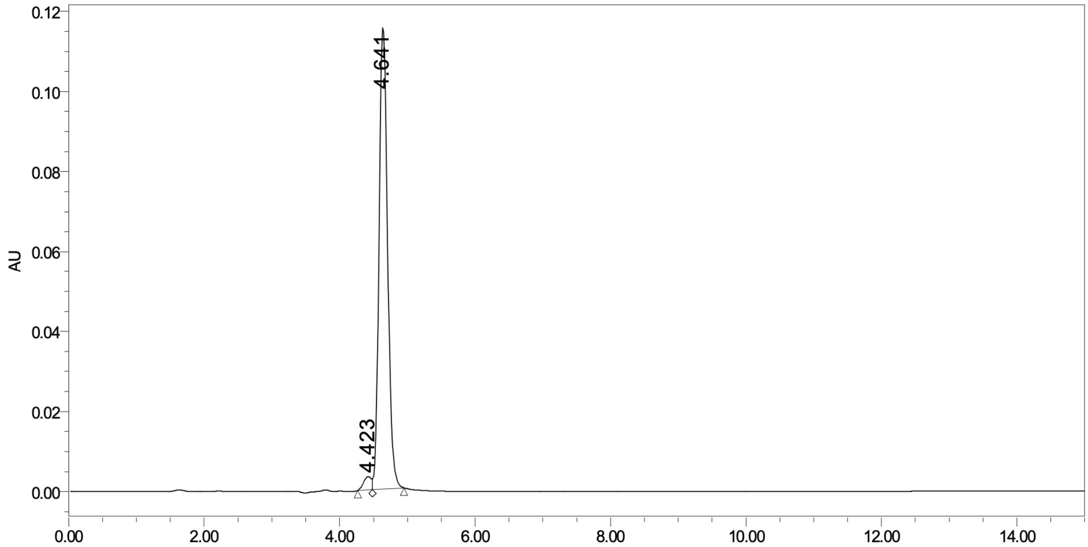

HPLC analysis of **ZZW-144** (Method 2)

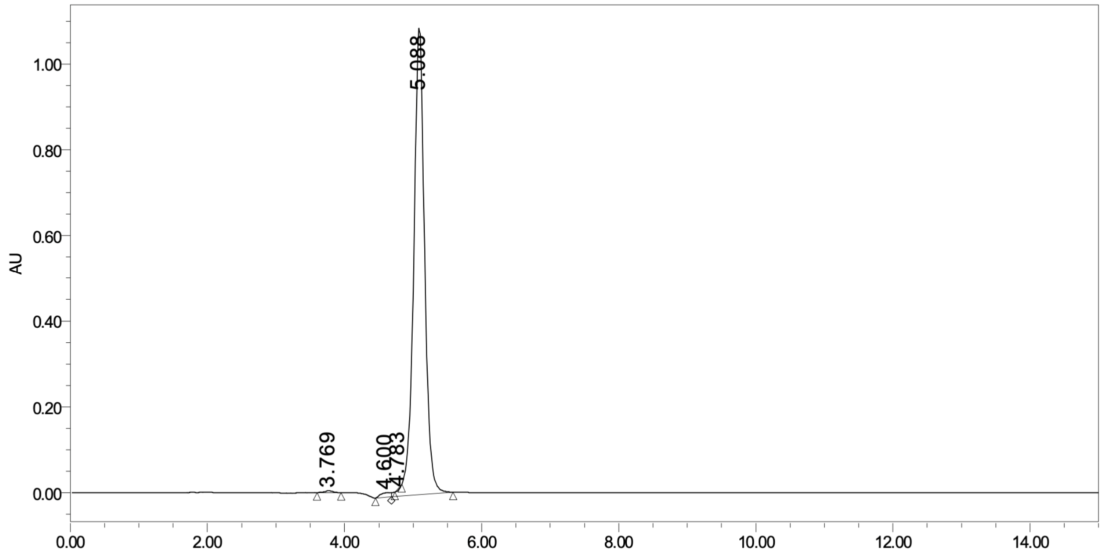

HPLC analysis of **ZZW-145** (Method 1)

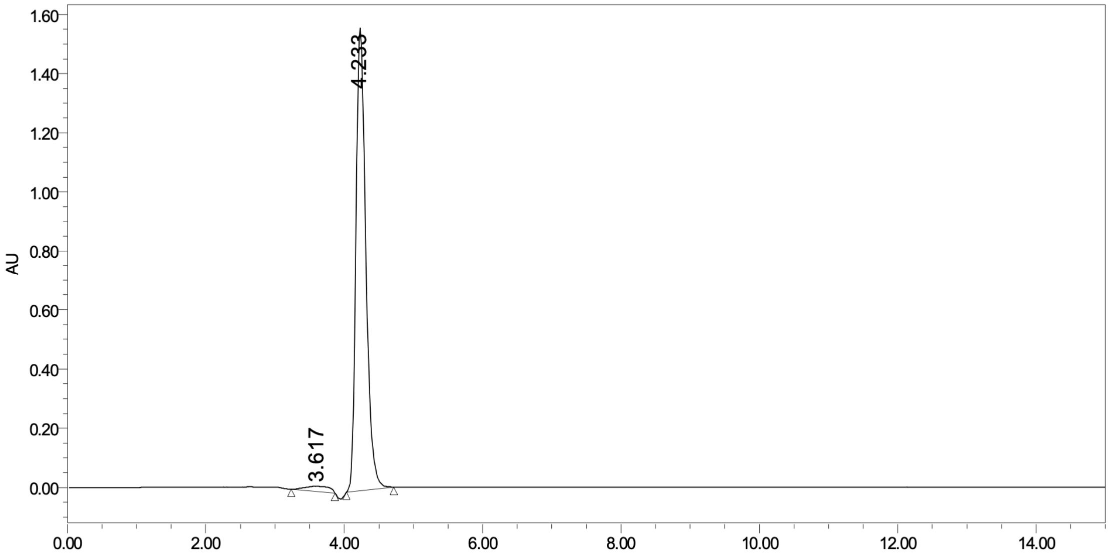

HPLC analysis of **ZZW-145** (Method 2)

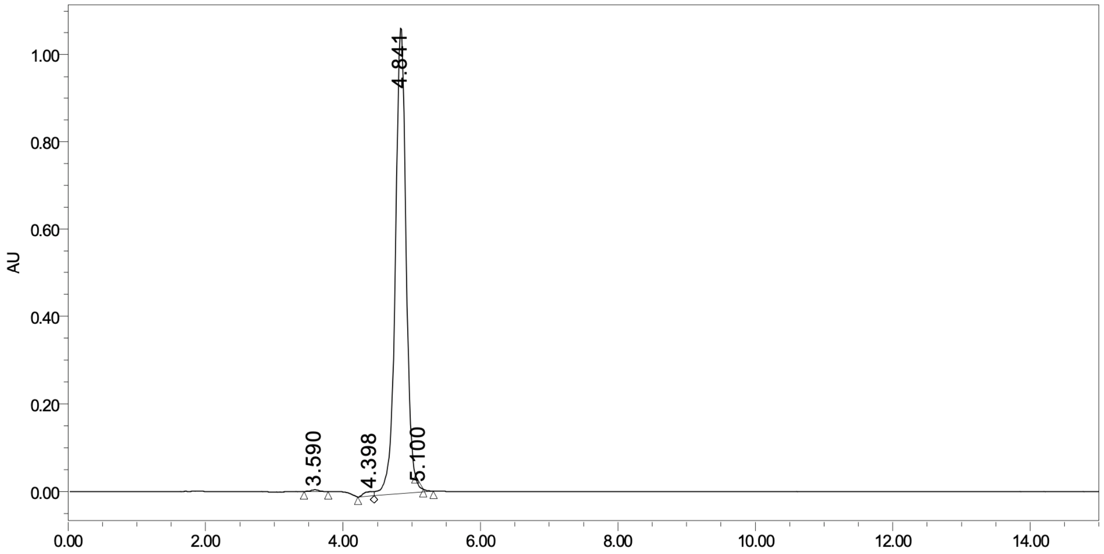

HPLC analysis of **ZZW-148** (Method 1)

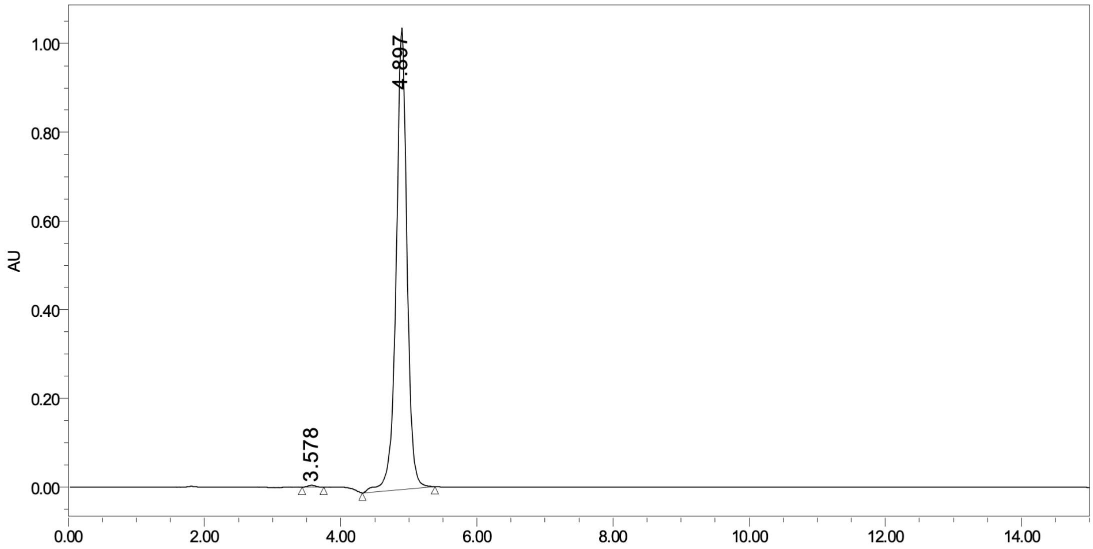

HPLC analysis of **ZZW-148** (Method 2)
